# Comparative Analysis on Polyphenolic Composition of Different Olive Mill Wastewater and Related Extra Virgin Olive Oil Extracts and Evaluation of Nutraceutical Properties by Cell-Based Studies

**DOI:** 10.3390/foods13203312

**Published:** 2024-10-18

**Authors:** Doretta Cuffaro, Andrea Bertolini, Ana Margarida Silva, Francisca Rodrigues, Daniela Gabbia, Sara De Martin, Alessandro Saba, Simone Bertini, Maria Digiacomo, Marco Macchia

**Affiliations:** 1 Department of Pharmacy, University of Pisa, 56126 Pisa, Italy; doretta.cuffaro@unipi.it (D.C.); simone.bertini@unipi.it (S.B.); marco.macchia@unipi.it (M.M.); 2 Interdepartmental Research Center “Nutraceuticals and Food for Health”, University of Pisa, 56100 Pisa, Italy; alessandro.saba@unipi.it; 3 Department of Surgical, Medical and Molecular Pathology and Critical Care Area, University of Pisa, 56126 Pisa, Italy; a.bertolini@med.unipi.it; 4 REQUIMTE/LAQV, ISEP, Polytechnic Institute of Porto, Rua Dr. António Bernardino de Almeida, 4249-015 Porto, Portugal; ana.silva@graq.isep.ipp.pt (A.M.S.); francisca.rodrigues@graq.isep.ipp.pt (F.R.); 5 Department of Pharmaceutical and Pharmacological Sciences, University of Padova, 351131 Padova, Italy; daniela.gabbia@unipd.it (D.G.); sara.demartin@unipd.it (S.D.M.); 6 Center for Instrument Sharing of the University of Pisa (CISUP), 56126 Pisa, Italy

**Keywords:** olive mill wastewater, polyphenols, nutraceutical, oleacein, antiinflammatory, by-products

## Abstract

This study reports a comparative analysis of the polyphenolic composition and nutraceutical properties of different olive mill wastewater (OMWW) and corresponding extra virgin olive oil (EVOO) extracts. Specifically, four OMWWs and corresponding EVOOs from cultivars Frantoio (A) and Leccino (B) obtained from different crushing seasons (early-stage (A1 and B1) and later-stage (A2 and B2)) were analyzed. Employing HPLC-DAD and LC-MS methods, the primary polyphenol content was identified and quantified. Overall, OMWW extracts showed a greater polyphenolic content compared to corresponding EVOO extracts, with OMWW B1 displaying the highest levels of polyphenols. The antiradical properties of extracts towards radical species (DPPH, ABTS, O_2_^−^, and HOCl^−^) were demonstrated in vitro, revealing a correlation with polyphenolic content. In fact, OMWW B1 and B2 demonstrated the strongest antiradical activity. Exploring nutraceutical properties of OMWWs, the intestinal permeation of the main polyphenols in a co-culture model (Caco-2 and HT29-MTX cell lines) was assessed, with tyrosol achieving a permeation of almost 60%. Furthermore, the involvement in the inflammation process has been evaluated in cell studies on THP1-derived macrophages by immunocytochemistry, demonstrating that OMWW B1 may exert an anti-inflammatory effect by modulating specific phenotype expression on macrophages. In conclusion, this study provides evidence supporting the reuse of OMWWs as a source of polyphenols with nutraceutical properties.

## 1. Introduction

The traditional Mediterranean diet entails consuming predominantly vegetables, legumes, and fruit, along with Extra Virgin Olive Oil (EVOO) as the primary source of dietary fat. EVOO is produced from fruits of the *Olea europaea* L. tree and is characterized by a unique flavor, highly appreciated among consumers [1,2]. The consumption of EVOO is associated with various nutraceutical benefits, comprizing a reduction in cardiovascular risk, neurological disorders, and breast cancer [3,4,5], due to its specific composition and, particularly, polyphenols. EVOO phenolic compounds encompass secoiridoids, lignans, phenyl alcohols, phenyl acids, and flavonoids. Among these, secoiridoids represent the most significant class of phenolic compounds in EVOO, being exclusively found in plants of the *Oleaceae* family. The most representative secoiridoids, in freshly produced EVOO, include the dialdehydic form of decarboxymethyl oleuropein aglycone (oleacein) and the dialdehydic form of decarboxymethyl ligstroside aglycone (oleocanthal). These bioactive compounds originate from the enzymatic hydrolysis of the glycoside forms of oleuropein and ligstroside, respectively. According to several studies, EVOO polyphenols have considerable health effects, spacing from chemopreventive and cardioprotective to neuroprotective properties [6,7,8].

The Mediterranean area is the principal producer of EVOO, due to favorable pedoclimatic conditions that guarantee a good quality of olives and, consequently, of EVOO. The EVOO production includes the crucial step of extraction that can be managed by a two or three-phase decanter process [9]. The three phase extraction is one of the most used systems, especially in Italy, and involves the addition of a significant amount of water (up to 50 L per 100 g of olive paste) [10]. The primary by-product of olive oil production is olive mill wastewater (OMWW), with an estimated annual production of 30 million cubic meters [11] that significantly impacts the environment due to management difficulties. Moreover, the high polyphenol content in OMWW disallows its use in irrigation processes due to its toxicity towards plants and microorganisms [12]. However, OMWW polyphenols are recognized as valuable bioactive compounds with notable nutraceutical properties, offering the potential for valorization in various applications.

OMWW extracts are noted as a plentiful source of distinct polyphenols. According to the literature, the main phenolic compounds found in OMWW include hydroxytyrosol, tyrosol, verbascoside, and various cinnamic acids, such as caffeic, gallic, vanillic, and syringic acids [13]. Among their various properties tyrosol and hydroxytyrosol [14] have shown the capacity to modulate the inflammatory response involved in different chronic diseases. For example, it has been reported that tyrosol and hydroxytyrosol counteract inflammation and oxidative stress, two hallmarks of hepatic fibrosis, a chronic liver disease caused by the excessive wound-healing response of the liver to persistent injury [15,16,17,18]. Their effect is mediated by modulation of the Th17/IL17A axis, as indicated by the reduction in IL6, IL17, and IL23 expression, suggesting that these two phenolic compounds may also modulate the immune hepatic microenvironment, in particular macrophages and regulatory T cells that are involved in fibrosis worsening [15].

While EVOOs are abundant in secoiridoid glucosides, OMWW exhibits high concentrations of secoiridoid derivatives, such as hydroxytyrosol, tyrosol, and occasionally oleacein and oleuropein [19]. Oleacein and oleuropein are secoiridoids primarily found in EVOO and olive leaves, respectively, though they may sometimes be present in OMWW. Available literature highlights the pharmaceutical potential of oleuropein and oleacein against various diseases such as obesity, cardiovascular complications, cancer, inflammation, and oxidation [20,21].

Nonetheless, to better understand the bioavailability of bioactive compounds and predict their effective nutraceutical effects, it is crucial to evaluate their pharmacokinetic properties, particularly their behavior in the digestive system. In fact, high permeation is inherently linked to absorption, from a regulatory perspective. In recent years, several studies have evaluated the intestinal permeability of bioactive compounds, including EVOO polyphenols, providing important information on the resulting health benefits [22,23,24]. Therefore, this work aims to investigate the polyphenolic composition and nutraceutical potential of OMWW derived from EVOO production. Considering that some studies have previously documented significant changes in the phenolic profile during the maturation of olive fruit across different cultivars [25], we selected four OMWW samples from traditional Tuscan cultivars (Leccino and Frantoio) obtained from three-phase oil mills during different periods of the crushing season (early and late stages).

A quali-quantitative HPLC and LC/MS analysis of the polyphenolic composition between the OMWWs and the corresponding EVOOs was examined to fully comprehend the variation in the polyphenol content. The antiradical properties of OMWW and EVOO extracts towards radical species (2,2-Diphenyl-1-picrylhydrazyl (DPPH), the 2,2-azinobis (3-ethyl-benzothiazoline-6-sulfonic acid) (ABTS)) were demonstrated in vitro, revealing a correlation with polyphenolic content. Moreover, the nutraceutical properties of OMWW were further explored, assessing the further antiradical properties (O^2−^ and HOCl^−^), and in vitro cytotoxicity. The intestinal permeation of the main OMWW polyphenols was investigated using a co-culture model (Caco-2 and HT29-MTX cell lines), two main cell types, absorptive and goblet cells, respectively, found in the small intestinal epithelium. Caco-2/HT29-MTX co-culture closely mimics the human intestinal epithelium to achieve more physiological conditions and, therefore, is useful for the investigation of transport over intestinal epithelium [26]. Furthermore, the contribution to the inflammation process was evaluated on THP1-derived macrophages evaluating the protein expression of the three differentiation markers CD68 (generic macrophage marker), CD86 (expressed in M1 proinflammatory macrophages), and CD163 (expressed in M2 anti-inflammatory macrophages) by immunocytochemistry. Our findings strongly support with multiple evidence, the utilization of polyphenol-rich OMWWs as an innovative, renewable, and low-cost source of bioactive compounds with different potential nutraceutical applications.

## 2. Materials and Methods

### 2.1. Chemistry and Reagents

Water, methanol (MeOH), acetonitrile (ACN), 2-propanol, and formic acid (FA, ≥98%) were LC-MS grade, while n-hexane, dichloromethane, ethanol (EtOH), ethyl acetate (AcOEt), and acetic acid (AcOH, 100%) were HPLC grade. All of them were from Merck (Merck KGaA, Darmstadt, Germany), as well as the commercial analytical standards of pinoresinol, p-hydroxyphenylacetic acid, and Trolox. Tyrosol, hydroxytyrosol, caffeic acid, vanillic, ferulic acid, p-coumaric acid, and vanillin were purchased from TCI (Zwijndrecht, Belgium), and the pure standards of oleocanthal and oleacein afforded by EVOO purification using the method described in our previous study [27].

Catechin, nitroetrazolium blue chloride (NBT), dihydrorodamine (DHR), and sodium hypochlorite (NaOCl) were purchased from Sigma-Aldrich, Germany. Dimethylformamide (DMF), disodium phosphate (Na_2_HPO_4_), and monopotassium phosphate (KH_2_PO_4_) were obtained from Merck, Germany. β-nicotinamide adenine dinucleotide (NADH) and phenazine methosulfate (PMS) were purchased from Sigma-Aldrich, India, and the USA, respectively. Caco-2 (clone type C2Bbe1) and HT29-MTX, were acquired from American Type Culture Collection (ATCC, DC, Washington, USA) and offered by Dr. T. Lesuffleur (INSERMU178, Villejuif, France), respectively. Dulbecco’s modified Eagle’s medium (DMEM) with GlutaMAX-I, fetal bovine serum (FBS), streptomycin, penicillin, and amphotericin B were from Invitrogen Corporation (Life Technologies, S.A., Madrid, Spain). Triton X-100 was purchased from Sigma Chemical Co. (St. Louis, MO, USA), while dimethylsulfoxide (DMSO) was obtained from AppliChem (Darmstadt, Germany). Roswell Park Memorial Institute (RPMI) medium was purchased from Corning (Corning, NY, USA). Phorbol 12-myristate 13-acetate (PMA) was purchased from Santa Cruz Biotechnology (Dallas, Texas, USA). 2,2-Diphenyl-1-picrylhydrazyl (DPPH) and (ABTS) were purchased from Merck (Darmstadt, Germany).

### 2.2. Sample Collection

EVOO and OMWW samples belong to two different Mediterranean cultivars (A: Frantoio and B: Leccino). The samples, produced by a three-phase oil mill, were collected in 2022 at two different times of crushing season: in early-stage October 2022 (A1 and B1) and in later-stage November 2022 (A2 and B2). EVOO samples (acid value < 0.5%, peroxide value < 10 meq O_2_/kg) were collected and immediately stored at −20 °C until further analyses. OMWW samples are part of the same EVOO production process and after collection were stored at −20 °C.

### 2.3. Samples Extraction

#### 2.3.1. EVOO

The EVOO extracts were prepared by liquid–liquid extraction as previously described by Esposito Salsano et al. [28]. Briefly, 3 g of EVOO was mixed with 12 mL of n-hexane and 15 mL of ACN. The resulting mixture was homogenized with a vortex mixer for 30 s and a rotary shaker for 30 min. Afterward, samples were centrifuged at 4000 rpm for 5 min and the ACN phase was collected and evaporated under reduced pressure to obtain the extract, which was stored at −20 °C or dissolved in 1 mL of MeOH/H_2_O (1:1, *v*/*v*) and analyzed by HPLC.

#### 2.3.2. OMWW

OMWW extracts were obtained by liquid–liquid extraction, according to the procedure described by Cuffaro et al. [13,29]. Briefly, 10 mL of OMWW were acidified to pH 2 with HCl 1N and n-hexane (15 mL) added to remove the lipid fraction, then shaken and centrifuged for 5 min at 3000 rpm. After separation, the treatment with n-hexane was repeated twice in succession. 10 mL of AcOEt were added to carry out the extraction of phenolic compounds and the mixture was vigorously shaken and centrifuged for 5 min at 3000 rpm. The phases were separated, and the extraction was repeated successively 3 times. Finally, AcOEt was evaporated under a vacuum to obtain the dry residue, being stored at −20 °C or dissolved in 1 mL of MeOH/H_2_O (1:1, *v*/*v*) and analyzed by HPLC.

### 2.4. HPLC Characterization

#### 2.4.1. EVOO

The EVOO extracts were analyzed in a Shimadzu HPLC Nexera series (model CBM-40D), constituted by a binary pump (LC-40D XR), a degassing unit (DGU-405), and a diode array detector (SPD-M40) (Shimadzu, OR, USA). For data processing, Shimadzu LabSolutions software LC-GC version 5.111 (Shimadzu, OR, USA) was used. At the stationary phase, a Phenomenex Gemini reverse-phase C18 column (250 mm × 4.6 mm, 5 µm particle size; Phenomenex, Castel Maggiore, Italy) was adopted and p-hydroxyphenylacetic acid was the internal standard. At the mobile phase, a mixture of H_2_O/AcOH (97.5:2.5, *v*/*v*) (A) and ACN/MeOH (1:1, *v*/*v*) (B) was used. The linear gradient is: 5% (B) to 30% (B) in 45 min; 70% (B) from 45 to 65 min; 80% (B) from 65 min to 70; 80% (B) from 70 to 85 min; 100% (B) from 85 min to 90 min; after re-equilibration in 5 min. The sample was injected (20.0 μL) as a mixture of MeOH/H_2_O (1:1, *v*/*v*). The flow rate was 1 mL/min.

#### 2.4.2. OMWW

The extracts were analyzed with the same Shimadzu HPLC mentioned in the Section 2.4.1. HPLC analysis was performed using a mixture of H_2_O/AcOH (99.75:0.25, *v*/*v*) (A) and MeOH/ACN (1:1, *v*/*v*) (B) as mobile phase, with a linear gradient elution condition as follows: 5% B (0–30 min), 30% (30–40 min) 98% (40–45 min), and 5% B (45–50 min). The sample was injected as a mixture of MeOH/H_2_O (1:1, *v*/*v*) concentration (100 mg/mL). The flow rate was 1 mL/min and the injected volume was 20.0 μL.

### 2.5. LC-MS/MS Instrumental Layout and Parameters

The instrumental setup consisted of an Agilent (Santa Clara, CA, USA) 1290 UHPLC system, which included a binary pump, a column oven maintained at 40 °C, and a temperature-controlled autosampler. This system was connected to a Sciex (Concord, ON, Canada) QTRAP 6500+ mass spectrometer operating as a triple quadrupole, equipped with an IonDrive™ Turbo V source performing electrospray ionization (ESI). Chromatographic separation was achieved using an Agilent Zorbax SB-C18 StableBond Analytical column (Santa Clara, CA, USA) with dimensions of 4.6 mm × 150 mm and a particle size of 5 μm. The mobile phases used were (A) MeOH and (B) H_2_O 100%, both containing 0.025% CH_3_COOH. A gradient elution method was employed at a flow rate of 800 μL/min, with the following conditions: 0.0–1.0 min (B) 100%, 12.0–14.0 min (B) 5%, 15.0–18.0 min (B) 100%. The injection volume was set to 10 μL. The instrument control and data acquisition were performed using Sciex Analyst^®^ version 1.7 software and MultiquantTM software version 3.0.3. Data analysis was carried out using Microsoft 365^®^ Excel software (Albuquerque, NM, USA) and GraphPad (San Diego, CA, USA) Prism version 9.0.2. The mass spectrometry selected reaction monitoring (SRM) method was operated in negative ion mode. For each compound, declustering potential (DP), collision energy (CE), and collision exit potential (CxP) were optimized, and three transitions were considered for analysis. One transition was used for quantification (Q), while the other two were used as qualifiers (q). The transition list and additional operational parameters are already reported [29].

A stock solution of each standard compound, including the internal standard, was prepared in MeOH at a concentration of 100 μg/mL and stored at −20 °C. Calibration curves were freshly prepared in pure LC-MS water [29]. Samples were thawed at room temperature (rt) and then added with the appropriate amount of the internal standard to achieve the same final concentration (500 ng/mL) in both the submitted samples and in the curve calibration levels.

### 2.6. Determination of Total Phenolic Content

The total phenolic content (TPC) of the extracts was assessed using the Folin–Ciocalteu reagent (FCR), following previously established protocols [30,31]. A total of 2 mg of dry extract was reconstituted in 1 mL of an 80% *v*/*v* methanol solution. Subsequently, 0.25 mL of FCR and 1.5 mL of sodium carbonate (Na_2_CO_3_) solution (20% *w*/*v*) were added to 1 mL of the methanolic extract in a volumetric flask. The solution was then diluted with distilled water to a final volume of 10 mL. The mixture was incubated at 25 °C for 45 min, after which the absorbance was measured at a wavelength of 725 nm. The TPC was quantified using a calibration curve constructed with gallic acid (concentration range: 2.5–40.0 µg/mL) and expressed as milligrams of gallic acid equivalent per kilogram of sample (ppm). Analyses were conducted in triplicate, and the mean value was calculated for each sample.

### 2.7. Antiradical Activity

#### 2.7.1. DPPH Assay

The antiradical activity of OMWW and EVOO samples was assessed by the DPPH free radical scavenging, as reported by Cuffaro et al. [29]. Briefly, 100 μL of DPPH MeOH solution (40.0 μg/mL) was added to 100 μL of the sample solution in MeOH (1 mg/mL to 0.01 mg/mL), and the resulting solution was incubated for 45 min at rt and in the dark. The absorbance was read at 517 nm in a Molecular Devices SPECTROstarNano (200–1000 nm) UV/Vis spectrophotometer. Trolox^®^ was the positive control and was treated under the same conditions as the samples. The percent of antioxidant activity (%AA) was calculated according to the following equation:

%AA = (AbsDPPH − Abssample/AbsDPPH) × 100

AbsDPPH = DPPH solution absorbance

Abssample = DPPH solution containing the test compound absorbance

The results were expressed as inhibitory concentration of 50% (IC_50_). All experiments were performed in triplicate.

#### 2.7.2. ABTS Assay

The ABTS free radical scavenging activity of samples was evaluated by radical cation colorimetric assay in accordance with Cuffaro et al. [29]. Briefly, the ABTS solution (7 mM of aqueous solution of ABTS with 2.45 mM potassium persulfate in a 1:1 ratio) was incubated for 12 h in the dark at rt. Then, the solution was diluted with EtOH to obtain an absorbance of 0.7 at 750 nm. A total of 10 μL of sample solution (1 mg/mL to 0.01 mg/mL) in EtOH was mixed with 180 μL of ABTS solution and the solution was incubated for 5 min at rt. The final absorbance was read at 734 nm in a Molecular Devices SPECTROstarNano (200–1000 nm) UV/Vis spectrophotometer. The % scavenging ability was calculated as follows:

% scavenging ability = (AbsABTS − Abssample/AbsABTS) × 100

AbsABTS = ABTS solution absorbance

Abssample = ABTS solution containing the test compound absorbance.

The percentage of scavenging ability was calculated against the sample concentration to obtain the inhibitory concentration at 50% (IC_50_). Trolox^®^ was employed as positive control and was treated under the same conditions as the samples. All experiments were performed in triplicate.

### 2.8. Reactive Oxygen Species Scavenging Capacity

#### 2.8.1. Superoxide Radical Scavenging Assay

The superoxide radical (O_2_^−^) scavenging capacity was spectrophotometrically evaluated, in accordance with Gomes et al. [32]. Absorbance was read at 560 nm for 6 min at 37 °C in a Synergy HT Microplate Reader. The results were expressed as IC_50_, of the reduction in NBT to a purple-colored diformazan by the reaction with O_2_.

#### 2.8.2. Hypochlorous Acid Scavenging Assay

The uptake capacity of hypochlorous acid (HOCl^−^) was determined by monitoring the effect of OMWW extracts in the HOCl^−^ induced oxidation of dihydrorhodamine (DHR) to rhodamine, as reported by Gomes et al. [32]. The fluorescence was read at 37 °C for 5 min, at wavelengths of 485 ± 20 nm and 528 ± 20 nm. The results were expressed as the inhibition, in IC_50_, of HOCl^−^ induced DHR oxidation.

### 2.9. Cell Viability Assay

Cell viability assays were performed in intestinal cell lines, namely Caco-2 and HT29-MTX. Passages 9–15 and 47–52 were, respectively, used for Caco-2 and HT29-MTX cells. The vital mitochondrial dye 3-(4,5-dimethylthiazol-2-yl)-2,5-diphenyltetrazolium bromide (MTT) assay was executed according to the methodology described by Pinto et al. [33]. Triton X-100 1% (*w*/*v*) and DMEM were used as negative and positive controls, respectively. Results were expressed as percentages of cell viability.

### 2.10. Intestinal Permeability Assay

The intestinal model, constituted by a co-culture of Caco-2 and HT29-MTX cell lines, was prepared following the method described by Araújo and Sarmento and by González et al. [34]. The experiments were performed 21 days after seeding the cells in 12 well plates. During this period, the transepithelial electrical resistance (TEER) was monitored to evaluate the cell monolayer integrity. On the last day, cell monolayers were pre-equilibrated with fresh HBSS (pH 7.4) at 37 °C for 30 min. Afterward, 0.5 mL of the sample solution in HBSS was added to the apical side of the co-culture monolayers while 1.5 mL of HBSS to the basolateral side. Samples were withdrawn from the receptor side at different timepoints (0, 15, 30, 45, 60, 90, 120, 150, 180, and 240 min) to establish the transport of bioactive compounds across the monolayer. At the same time, the TEER was evaluated, using an EVOM Epithelial Volthometer Instrument equipped with a chopstick electrode (World Precision Instruments, Sarasota, FL, USA). After each sampling time, the basolateral side was replaced with the same HBSS volume. Samples were conserved at −20 °C for subsequent LC/ESI-MS analysis, according to Section 2.5.

### 2.11. Immunocytochemistry on THP-1-Derived M0 Macrophages to Assess CD86 and CD163 Expression

THP-1 cells were maintained in an RPMI complete medium, containing L-glutamine (1%), streptomycin/penicillin (1%), and 10% FBS, at 37 °C with 5% CO_2_ in a humified atmosphere. To induce their differentiation into M0 macrophages, able to polarize into specific macrophage subtypes, THP-1 cells were seeded on a glass coverslip into a 24-well plate (105 cells/mL) and treated with 320 nM PMA for 24 h. Cells were then treated for 24 h with OMWWs at a concentration of 6.25 μg/mL in a complete medium. Their effect on macrophage polarization was assessed by means of immunocytochemistry, evaluating the protein expression of CD86 and CD163, markers of polarization to M1-like or M2-like phenotype, respectively [35]. Briefly, cells were fixed with 4% PFA, PBS-washed, and permeabilized for 5 min with 0.1% Triton X-100. Fixed cells were incubated with rabbit anti-CD86 or anti-CD163 (1:500) primary antibodies at 4 °C overnight and then, with AlexaFluor647 goat anti-rabbit IgG secondary antibody (1:200) in the dark for 1 h at rt. Hoechst33342 (1:400) was used as a nuclear counterstain. The images were acquired using a Zeiss LSM800 confocal microscope and quantified with the Image J software version 2.9.0 (LOCI, Madison, WI, USA). Mean fluorescence intensity was plotted and analyzed.

### 2.12. Statistical Analysis

All measurements were performed in triplicate and the results were presented as mean ± standard deviation (SD) (n = 3). One-way ANOVA test, followed by the HSD Tukey test was employed to perform the data analysis, through the IBM SPSS Statistics 27.0 software (SPSS Inc., Chicago, IL, USA) or GraphPad Prism software ver. 10.2.

## 3. Results and Discussion

The health-promoting properties of EVOO are well recognized, due to the presence of different nutraceutical compounds, particularly polyphenols [7,36,37]. EVOO production can adopt different extraction methods, with the three-phase technique as the most commonly used. This system includes a fundamental centrifugation step that completely separates EVOO from OMWW, its main by-product. OMWWs are a significative source of environmental pollution due to their high content of chemicals and biochemical oxygen demand that confers a strong acidity [38]. Nevertheless, a considerable fraction of polyphenols is conserved in OMWWs.

Indeed, olive fruits are generally rich in polyphenols and only a small percentage, 1–2%, of the total phenolic content, is transferred to EVOO. The resulting 98% of polyphenols are distributed during EVOO production: 53% in OMWW and 45% in pomace [39,40,41]. The addition of water during the olive oil centrifugation process is the main reason why in EVOO polyphenols are slightly recovered compared to OMWW. In fact, the high polyphenol content of OMWW, as already reported in the literature, was mainly due to the partition coefficient (oil/water) of most of the olive biophenols in favor of the water phase [13], which makes OMWW a consistent source of soluble polyphenols.

### 3.1. Analysis of Polyphenolic Contents

In this work, the polyphenolic composition of four extracts of OMWWs and the corresponding four extracts of EVOOs were analyzed by HPLC-DAD, and data are summarized in Table 1.

The compound identification was based on the direct comparison with standards and further confirmed by MS/MS fragmentation patterns. Qualitative HPLC-DAD chromatograms of all the samples are reported in Supporting info (Figures S1–S8).

**Figure 1:**
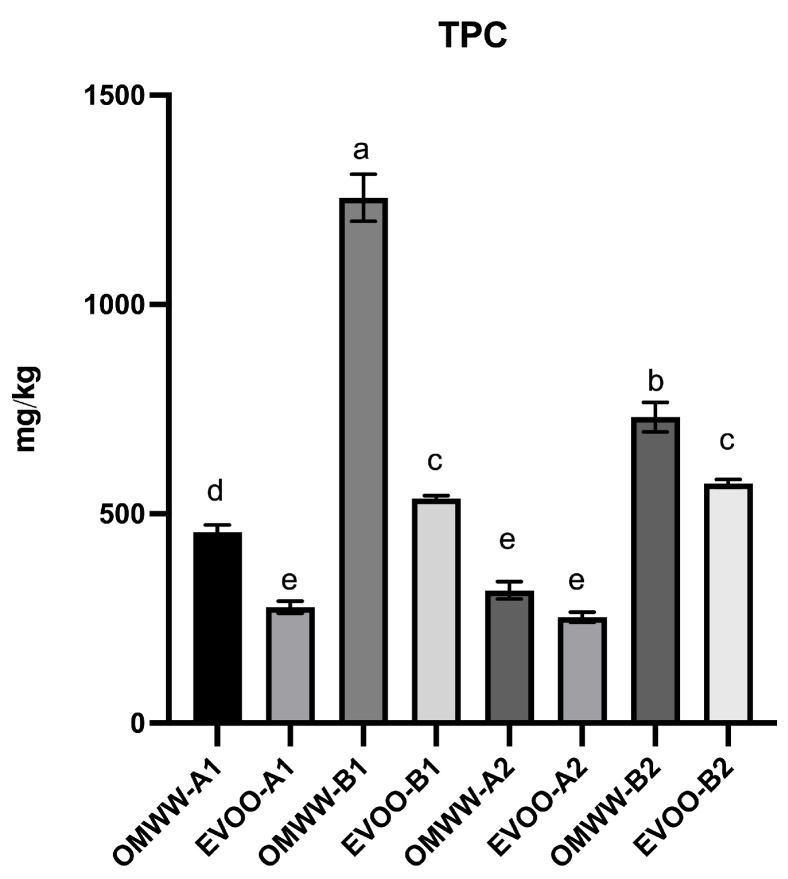
Total phenolic content (TPC, mg/kg) of EVOO and OMWW extracts. Values are expressed as mean ± standard deviation (n = 3). Different letters (a, b, c, d, e) in the same sample represent significant differences (*p* < 0.05) between different concentrations, according to Tukey’s HSD test.

As reported in Table 1 and Figure 1, in general, OMWW extracts contain higher total polyphenolic content (TPC) (mg/kg of OMWW) compared to the related EVOO extracts (mg/kg of EVOO). In particular, OMWW extracts deriving from the Leccino cultivar (B1 and B2) exhibited the highest concentration of total phenols (1255.4 mg/kg of OMWW and 730.8 mg/kg of OMWW, respectively) (Figure 1).

HPLC analysis revealed differences in the phenolic content between OMWW and EVOO, consistent with previously reported data [13,19,42]. As already demonstrated in other OMWW extracts [13,29,43,44,45], hydroxytyrosol is one of the most representative phenols resulting from the hydrolysis of oleuropein and oleacein, highly concentrated in olive fruits. In our OMWW samples, hydroxytyrosol ranges from 92.4 mg/kg for OMWW A1 to 339.0 mg/kg for OMWW B2. The OMWW content analysis revealed several other phenols, including simple phenols like tyrosol, phenolic acids such as caffeic acid and vanillic acid, secoiridoids such as oleuropein and oleacein and other polyphenols verbascoside, pinoresinol, and apigenin 7-glucoside as the main representative. Notably, the concentration of hydroxytyrosol, tyrosol, caffeic acid, and vanillic acid increases in all late-stage OMWW extracts when compared to early-stage OMWW extracts. Other polyphenols, such as verbascoside, oleuropein, pinoresinol, apigenin-7-glucoside, and oleacein, are present in lower amounts or even absent. Regarding OMWW A2, none of these polyphenols are detectable, whereas in OMWW B2, only oleuropein and oleacein are absent, and the other compounds (verbascoside, pinoresinol, and apigenin-7-glucoside) are reduced. It is worth noting that, with the tyrosol exception, these phenols are not typically found in EVOOs, despite being highly reported in olives [46]. This is due to the fact that during the olive processing, olive polyphenols were partitioned between EVOO and OMWW. The addition of a substantial amount of water results in a decrease of EVOO phenols owing to their hydrophilic nature that addresses their recovery in the water phase of OMWW [39].

In terms of the polyphenolic composition the EVOO examined in this study distinguishes them as EVOO possessing health benefits, adhering to the European Food Safety Authority (EFSA) health claim for olive oil containing at least 250 mg/kg of this polyphenols (hydroxytyrosol and its derivatives) [47]. In EVOO samples, notable concentrations of the secoiridoids oleocanthal (ranging from 216 µg/g to 379.6 mg/kg for EVOO A2 and EVOO B2, respectively) and oleacein (varying between 29.2 mg/kg to 180.7 mg/kg for EVOO A2 and EVOO B2, respectively) were quantified, in agreement with literature findings for fresh EVOOs [28,47]. Consequently, hydroxytyrosol and tyrosol were detected in minimal quantities (maximum 4.15 mg/kg for EVOO A2 and 10.6 mg/kg for EVOO B2), because typically resulted from the degradation of secoiridoids (oleacein and oleocanthal respectively) as already mentioned. The absence of oleocanthal in OMWW samples further confirmed this process, as OMWW extracts are richest in tyrosol.

Conversely, early-stage OMWWs showed a significant amount of oleacein, in particular in OMWW B1 (672.6 mg/kg). However, oleacein is not present in the later-stage samples (OMWW A2 and OMWW B2), likely due to its poor stability. Oleacein is rarely found in OMWW samples, and different papers, characterizing OMWW phenolic composition, have not reported the identification of this polyphenol. Recently, the detection of oleacein in OMWW composition has been discussed and specifically analyzed [48]. Carrara et al. detected oleacein in different French cultivars of olive with an innovative UPLC-MS-UV and HPLC-Fluorescence. In this work, oleacein varied in concentration from 0 to 60 mg/g. Similarly, Silvan et al. and Cuffaro et al. identified oleacein as the predominant compound at 21.1 mg/g and 314 mg/g, respectively [29,49].

The discrepancy in the quantification of oleacein in OMWW is strictly related to the variable concentration in olive fruits. In fact, several authors have observed that oleacein content increases during fruit ripening [50]. Similarly to our results, Sivakumar et al. noted the complete absence of oleacein in certain olive cultivars, and an improved quantification in specific cultivars during the months of October [51].

**Figure 2:**
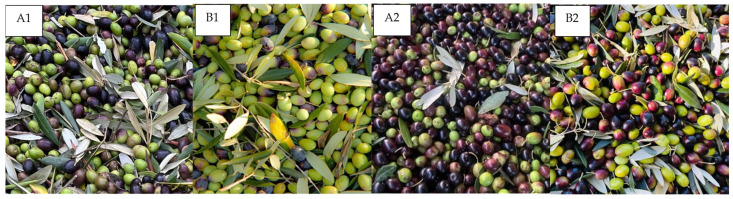
Evaluation of maturation stage of olives during the different time collections. (**A1**). olive cultivar Frantoio early-stage. (**B1**). olive cultivar Leccino early-stage. (**A2**). olive cultivar Frantoio later-stage. (**B2**). olive cultivar Leccino later-stage.

In agreement with these findings, in our study also the polyphenol concentrations in EVOO and OMWW varied significantly depending on both the cultivar and the timing of the olive harvest. Specifically, samples collected during the early harvest stage (A1 and B1) exhibited substantially higher polyphenolic content compared to those collected during the later harvest stage (A2 and B2). This increase in polyphenolic content is likely attributable to the green maturation stage of the olives harvested in October (Figure 2).

Olive fruits undergo two maturation stages, green and black, which are typically associated with higher and lower polyphenolic contents, respectively [52]. During the first collection (A1 and B1 samples), the olives were mainly at the green maturation stage (Figure 2), resulting in an increased polyphenol content compared to the second collection in November 2022 (A2 and B2 samples) where the olives were mostly maturated to the black color (Figure 2). This difference underscores the impact of the maturation stage on the polyphenolic content of the olives, which in turn influences the bioactive properties of the derived EVOO and OMWW.

Moreover, analyzing the difference in TPC due to cultivar, EVOOs from the Leccino cultivar (EVOO B1 and B2) contain higher levels of polyphenols compared to those from the Frantoio cultivar (EVOO A1 and A2) (Table 1 and Figure 1). Notably, as reported by Cecchi et al., the reduction in polyphenols due to the time of olive harvest is negligible for the Leccino cultivar, but evident for the Frantoio cultivar [53]. The concentration of phenols in olive oil is closely linked to the time of olive harvest, as each cultivar can reach maturity at different times during the growing season [53]. Similarly to EVOO, the OMWW polyphenols content is mainly linked to cultivar and the time of olive harvest. In fact, OMWWs Leccino (OMWW B1 and B2) possess a higher amount of polyphenols than OMWWs Frantoio (OMWW A1 and A2) (Table 1 and Figure 1).

### 3.2. Antiradical Properties

The evaluation of the extract’s antiradical properties is extremely relevant since the oxidation processes and the radical production are crucial for the development of different pathologies.

In the present study, the antiradical/antioxidant capacity of the OMWW and EVOO extracts was evaluated using a panoply of standard assays such as radical scavenging activity DPPH and ABTS. The DPPH and ABTS assays are fast and sensitive methods for evaluating the antioxidant/antiradical activity of various compounds by measuring their ability to scavenge free radicals. Table 2 summarizes the antiradical properties of OMWW and EVOO extracts. As can be observed, in general, OMWW extracts exhibited the strongest antioxidant effects compared to EVOO due to the higher polyphenol content and the specific polyphenol composition rich in phenolic acid.

For what concerns to the DPPH assay, OMWW A1 and OMWW B1 (early-stage IC_50_ = 0.79 mg/mL and IC_50_ = 0.16 mg/mL, respectively) possess an improved antiradical effect than the corresponding EVOOA1 and EVOOB1 (EVOO A1 IC_50_ = 1.16 mg/mL and EVOO B1 IC_50_ = 0.47 mg/mL). On the contrary, for the later-stage samples, OMWW A2 (IC_50_ = 0.77 mg/mL) and OMWW B2 (IC_50_ = 0.26 mg/mL), the IC_50_ are two times lower than those of the corresponding EVOO A2 and EVOO B2 (IC_50_ = 1.61 mg/mL and IC50 = 0.58 mg/mL, respectively). Regarding the ABTS assay, the antiradical activity of all OMWW extracts is better than EVOOs. The activity trend was maintained with all OMWW extracts being more active when compared to related EVOO. For both assays, the best results were achieved by the OMWW B2 sample. In general, the antiradical profile of the OMWW extracts is in agreement with other OMWW extracts reported in the literature [43,54].

These results clearly demonstrate a correlation between the higher polyphenolic content of OMWW samples when compared to the corresponding EVOOs, along with the preliminary screening of the antiradical activity evaluated by DPPH and ABTS assays. The correspondence between the antiradical capacity and TPC may be attributed to the high levels of tyrosol, as well as hydroxytyrosol, and phenolic acids, which are strong antiradical polyphenols in the OMWW extracts. Compounds with higher hydroxylation of the aromatic ring such as hydroxyl tyrosol which has a 3,4 dihydroxyl structure bonded to an aromatic ring usually proved high radical scavenging activity compared to tyrosol, for example, which has a similar structure, but with only one hydroxyl group bound to an aromatic ring. The antioxidant activity of phenolic acids was also related to the number of hydroxyl groups [55]. Therefore, caffeic acid was found to be a more potent antioxidant reporting a tri-hydroxylic group in its aromatic ring [56].

Overall, B samples display greater activity than A samples both for OMWW and EVOO, with the B cultivar showing strong antiradical polyphenolic extracts and elevated polyphenol content, too. The B2 sample proves to be the most active antiradical among all samples.

### 3.3. In Vitro Reactive Oxygen Species Scavenging Capacity

The reactive species formation is a mechanism involved in various human physiological processes, including homeostasis, inflammation, and cellular signaling [57,58]. Oxidative stress occurs when there is an imbalance between the antioxidant defense capacity of cells (e.g., enzymes, namely catalase and superoxide dismutase, and nonenzymatic antioxidants, such as ascorbic acid, glutathione, and α-tocopherol) and the generation of pro-oxidant reactive species, leading to the overproduction of reactive oxygen species (ROS) as well as reactive nitrogen species (RNS) [58]. Pro-oxidant reactive species are implicated in several chronic diseases, causing lipid oxidation, protein oxidation, and DNA damage, which can have detrimental effects on major cellular components. Oxygen (O_2_) is the primary source of ROS, which also includes less reactive species, such as hydrogen peroxide (H_2_O_2_), as well as more potent and toxic species, such as hypochlorous acid (HOCl^−^). Table 3 summarizes the scavenging capacity of OMWW extracts against O_2_^−^ and HOCl^−^.

Overall, the OMWW extracts showed good scavenging abilities reporting IC_50_ < 134 μg/mL for all the samples analyzed, probably due to their phenolic profile, including tyrosol, hydroxytyrosol, and especially phenolic acid, and verbascoside, whose scavenging power was already described by several authors [59,60]. Notably, OMWW B samples were revealed to be better scavengers for O_2_^−^ and HOCl^−^ radical species when compared to A samples, achieving IC_50_ ranging from 52 to 96 μg/mL. This result is strictly connected with the high polyphenolic content of OMWW B compared to A samples, and to the DPPH and ABTS assays, revealing also in this case that OMWW belong to cultivar Leccino are rich in antiradical polyphenols compared to cultivar Frantoio. OMWW B1 presented the best antiradical scavenging capacity, possibly due also to the richness in the secoiridoid oleacein, a well-recognized nutraceutical polyphenol with high in vitro antioxidant effect as reported by Czerwinska et al. In this study, oleacein demonstrated low IC_50_ in the inhibition of important radical scavengers such as O_2_^−^, H_2_O_2,_ and HClO among others [61].

### 3.4. In Vitro Cell Studies of OMWW Extracts Toward Caco-2 and HT29-MTX Intestinal Cell Lines

**Figure 3:**
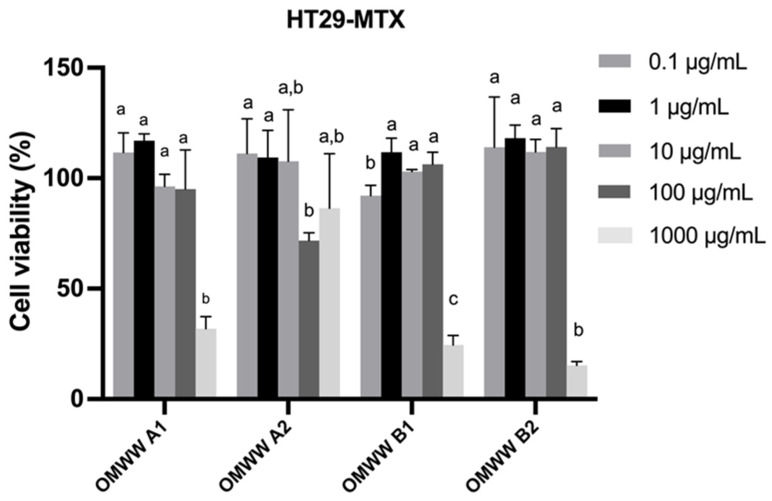
Effects of OMWW extract exposure on the viability of HT29-MTX cell line at different concentrations, as measured by the MTT assay. Values are expressed as mean ± standard deviation (n = 3). Different letters (a, b, c) in the same sample represent significant differences (*p* < 0.05) between different concentrations, according to Tukey’s HSD test.

**Figure 4:**
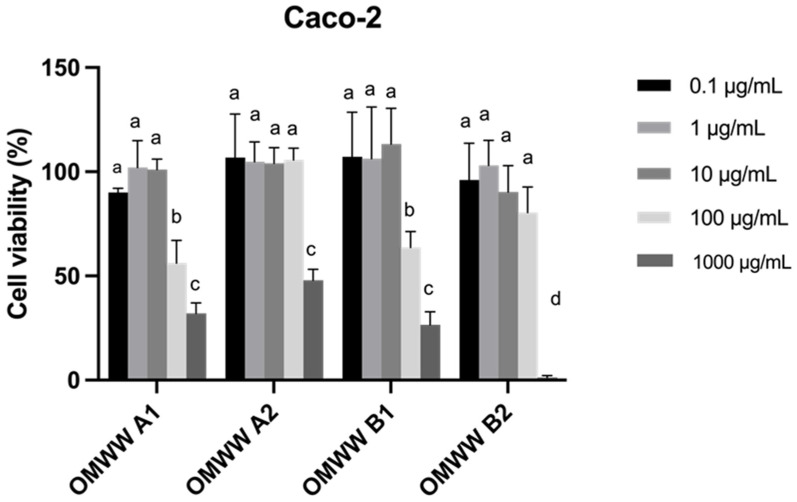
Effects of OMWW extract exposure on the viability of Caco-2 cell line at different concentrations, as measured by the MTT assay. Values are expressed as mean ± standard deviation (n = 3). Different letters (a, b, c, d) in the same sample represent significant differences (*p* < 0.05) between different concentrations, according to Tukey’s HSD test.

In vitro, cell model assays have been increasingly used to evaluate the toxicity of new nutraceutical ingredients. Considering the potential use of OMWW extracts in the nutraceutical field, the cytotoxicity evaluation on the most representative intestinal cell lines (Caco-2 and HT29-MTX) is crucial. The results of cell viability evaluated by MTT assay are shown in Figure 3 and Figure 4.

Based on the obtained results, the OMWW extracts did not significantly impact the viability of both cell lines at concentrations below 10 μg/mL, maintaining viabilities between 90% and 100%, while viabilities of 100% were observed for the tested concentrations of 0.1 μg/mL and 1 μg/mL of extract. At a concentration of 100 μg/mL, all the extracts are safe in HT29-MTX cell lines (viability > 80%), whereas only OMWW A2 and B2 maintain a vitality > of 80% for the Caco-2 cell line. However, the highest tested concentration (1000 μg/mL) significantly reduced the viability of both cell lines (1.35–47.9% in Caco-2 and 15–86% in HT29-MTX). On the other hand, at the highest concentration tested (1000 μg/mL), the viability decreased (15–30%) in the HT29-MTX cell line for all samples, except OMWW A2. In what concerns to the Caco-2 cell line, at 1000 μg/mL, the viability after exposure to OMWW B2 was only 1.35%, while ranging from 26% to 45% for the other samples. Considering the results obtained for both cell lines, the optimal non-cytotoxic concentration was inferior to 10 μg/mL. Literature reported similar data for the exposition of OMWW extracts in human cell lines, such as HUVECs (Human umbilical vein endothelial cells) and HSMCs (Human Mesenchymal Stem Cells), indicating the absence of toxicity up to 100 µg/mL [62]. To the best of our knowledge, this is the first investigation into the effects of OMWW extract on the Caco-2 and HT29-MTX intestinal cell lines.

### 3.5. Intestinal Co-Culture Model Permeation Assay

The bioaccessibility of nutrients, defined as the amount of nutrients released from the food matrix into the gastrointestinal tract, has a significant impact on bioactivity and bioavailability. It is widely recognized that polyphenols may bind to indigestible constituents, such as dietary fiber, or be metabolized into smaller molecules that are absorbed in the small intestine [63]. Several in vitro cell models have been developed to accurately assess the phenolic permeability across the intestinal barrier [22]. In particular, in vitro intestinal models that mimic the human mucosa at the absorption level are crucial to interpreting these dynamic and complex mechanisms [23]. In recent years, intestinal in vitro models have been mainly composed of a co-culture of different cells of the intestinal epithelium, particularly Caco-2 and HT29-MTX [26]. Caco-2 cells simulate the human colon due to the presence of microvilli and tight junctions as well as various transporters, enzymes, and nuclear receptors [23,24], while HT29-MTX cells can simulate the goblet cells, allowing evaluation of the mucoadhesion of carrier systems [24]. Therefore, this co-culture system is able to mimic the human intestinal epithelium, creating more physiologically relevant conditions and representing a useful model for investigating transport mechanisms across the intestinal epithelium [26].

In this study, the intestinal permeability of the polyphenols present in the OMWW B1 sample was assessed using a well-established co-culture model consisting of Caco-2 and HT29-MTX cells. OMWW B1 was selected as the most representative extract because it contains the most comprehensive variety of detected polyphenols at high concentrations.

**Figure 5:**
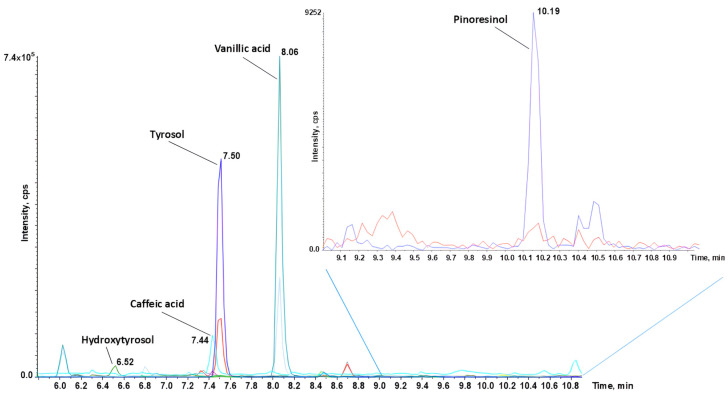
MRM chromatogram of detected polyphenols in a representative sample. For each analyte, multiple reaction monitoring (MRM) transitions/fragmentations were monitored: the one with the higher signal-to-noise ratio (S/N), usually corresponding to the most intense trace, was used for the quantification of the analyte (Q), while the other transitions, typically of lower intensity, were used to confirm that the peak is attributable to the analyte (q). Both transitions are necessary to ensure accurate quantification and detection of the compounds of interest. In this figure, two transitions per peak were extracted from the ion chromatogram (XIC) and associated with each specific target and its retention time. Chromatograms were obtained using Sciex Analyst^®^ software (version 1.7).

For each analyte, multiple reaction monitoring (MRM) transitions were monitored. The transition showing the highest signal-to-noise ratio, typically corresponding to the most intense trace, was used for quantification of the analyte, while the other transitions confirmed that the peak could be attributed to the analyte. A representative chromatogram, from a sample, is shown in Figure 5.

Specificity was assessed by repeatedly injecting analytes into the system and monitoring their retention times to ensure differentiation from other components and potential interferents present in the samples. Linearity was evaluated within the calibration curve range established previously. The concentrations of analytes were accurately and precisely determined by incorporating the internal standard in each sample and calibration curve point. This standard corrected for any errors or incidents that may have occurred during the pre- and analytical processes.

**Figure 6:**
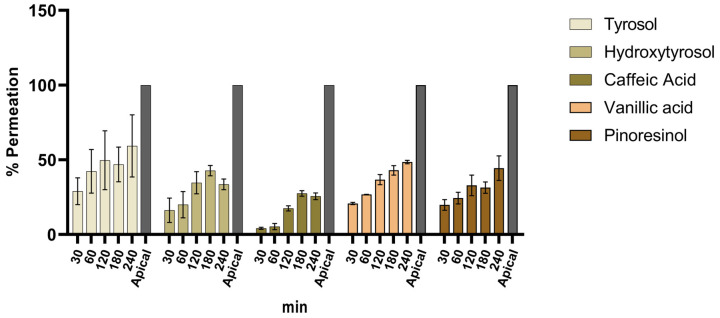
Permeation (%) of detected polyphenolic compounds from the B1 OMWW sample (500 µg/mL) at 30, 60, 120, 180, and 240 min through the intestinal model (n = 3). The bar graph uses different colors for each polyphenol to indicate the permeability rate achieved at different time points by the polyphenols detected within the OMWW sample dissolved and used for the permeation assay. The gray bar identifies the maximum achievable percentage permeability, represented by the apical side of the intestinal layer, from which the experiment begins.

Figure 6 summarizes the permeation percentage reached by each detected compound between 30 and 240 min analyzed by LC/MS analysis and Table 4 presents the percentage rates for all detected polyphenols. Raw concentrations calculated for each timepoint are reported in Supplementary Table S1.

Several compounds, among the most abundant in OMWW extract, and belonging to different families of polyphenols were correctly detected and quantified: tyrosol and hydroxytyrosol, the major components of the phenyl alcohols family, caffeic acid, member of the hydroxycinnamic acids, vanillic acid, part of the m-methoxybenzoic acids, and eventually pinoresinol, a furanoid lignan. The permeation results were expressed as the percentage of compound release, which was calculated by comparing the concentration on the apical side (100%) to the compound percentage that permeated through the model from the apical side (t = 0 min) to the basolateral side (t = 240 min).

The exposure of the OMWW B1 sample revealed distinct permeation rates for various polyphenols over a 240-min timeframe. Notably, tyrosol and vanillic acid achieved the highest permeation rates, reaching values of 59% and 48%, respectively, after 240 min. Pinoresinol also demonstrated a significant permeability rate, securing 44% at the same time point.

In contrast, hydroxytyrosol and caffeic acid displayed a lower permeation ability, with rates of 34% and 26%, respectively. The entrapment of certain compounds within intestinal cells, particularly under oxidative stress conditions, can explain the permeation rates observed for these compounds. In fact, cells under oxidative stress are more likely to absorb bioactive compounds with antioxidant properties, such as hydroxytyrosol and caffeic acid, which have proven to be effective antioxidants in human colon cell lines like Caco-2 and HT29-MTX [64,65]. Furthermore, the intracellular environment is notably different from the extracellular environment, which can lead to the metabolism of compounds within the cells. This intracellular metabolism may prevent the compounds from being detected in permeation samples, thereby contributing to the observed low permeation rates [66]. For instance, the absence of oleacein may be attributed to its potential degradation under oxygen and light exposure, causing metabolic instability [20], despite its substantial concentrations in the extract. The transport of bioactive compounds in the gastrointestinal tract can be influenced by a variety of factors, such as concentration, molecule size, polarity, and degradation processes. As evidenced by empirical studies [67], oleacein has the capacity to generate hydroxytyrosol subsequent to the phase I and phase II metabolism processes. Hydrolysis, a commonly observed phenomenon in phase I metabolism, is closely associated with diverse mechanisms of action. Among these mechanisms, carboxylesterases are a class of enzymes that possess the ability to catalyze the hydrolysis of esters, amides, and carbamates, resulting in the formation of corresponding carboxylic acids and alcohols.

When administered alone or in a complex matrix, as an OMWW sample, the permeation rates can change, and structurally simple compounds can report different, and even lower, percentages of permeation [68]. It is hypothesized that the presence of multiple polyphenols can induce competition processes in the cellular intestinal model hardening the achievement of their relative top, previously observed, permeation index [68].

However, the results obtained in this study show comparable phenolic compound permeation rates with other authors that used food-derived matrices and extracts [24]. To the best of our knowledge, this is the first study to assess the permeation ability of various polyphenols derived from olive oil and olive waste using an intestinal model. In a previous study [22], we investigated the in vitro intestinal permeation of tyrosol, utilizing the same model, and observed a high permeation rate, with 78% release at the final time point (240 min). These findings are consistent with the present results, where tyrosol, even within a complex matrix, exhibited a permeation rate of up to 60% after 240 min. The results obtained in this study have to be considered only as a preliminary approach since the gastrointestinal digestion process can influence and modulate the final bioavailability of phenolic compounds, where phenolic matrix conditions this physiological process [69]. These findings make valuable insights into the intestinal transport dynamics of specific polyphenols, shedding light on their bioavailability and potential health implications, giving important information for the valorization of OMWW extract in dietary supplements. Our results achieve fundamental information. Further investigations could delve into the molecular mechanisms governing the observed permeation variations among polyphenolic compounds, enhancing the understanding of their interaction with the intestinal barrier and the faith they encounter after the actual permeation.

### 3.6. In Vitro Effect of OMWWs on the Polarization of THP-1-Derived Macrophages

The positive outcomes regarding antioxidant and antiradical activities justified a further analysis of other nutraceutical properties of OMWWs, evaluating their involvement in inflammation in the THP-1 macrophage cellular model [70]. Macrophages are plastic cells that, after being polarized into multiple functional phenotypes under different pro-inflammatory or anti-inflammatory stimuli, may exert opposite roles in many human diseases, including infections and cancer. The two main subgroups are traditionally defined as M1-like macrophages, whose differentiation is promoted by interferon or lipopolysaccharide, with pro-inflammatory functions, and M2-like, induced by IL-4 and IL-13, which are in turn considered anti-inflammatory macrophages with protective features, being for example involved in tissue regeneration [71]. M0 are plastic, undifferentiated macrophages that may be further polarized into multiple functional phenotypes. Among them, the two most studied subgroups are defined as M1-like, CD86-expressing pro-inflammatory macrophages, whose differentiation is promoted by interferon or lipopolysaccharide, and M2-like, CD163-expressing anti-inflammatory macrophages that are induced by IL-4 and IL-13 [71].

**Figure 7:**
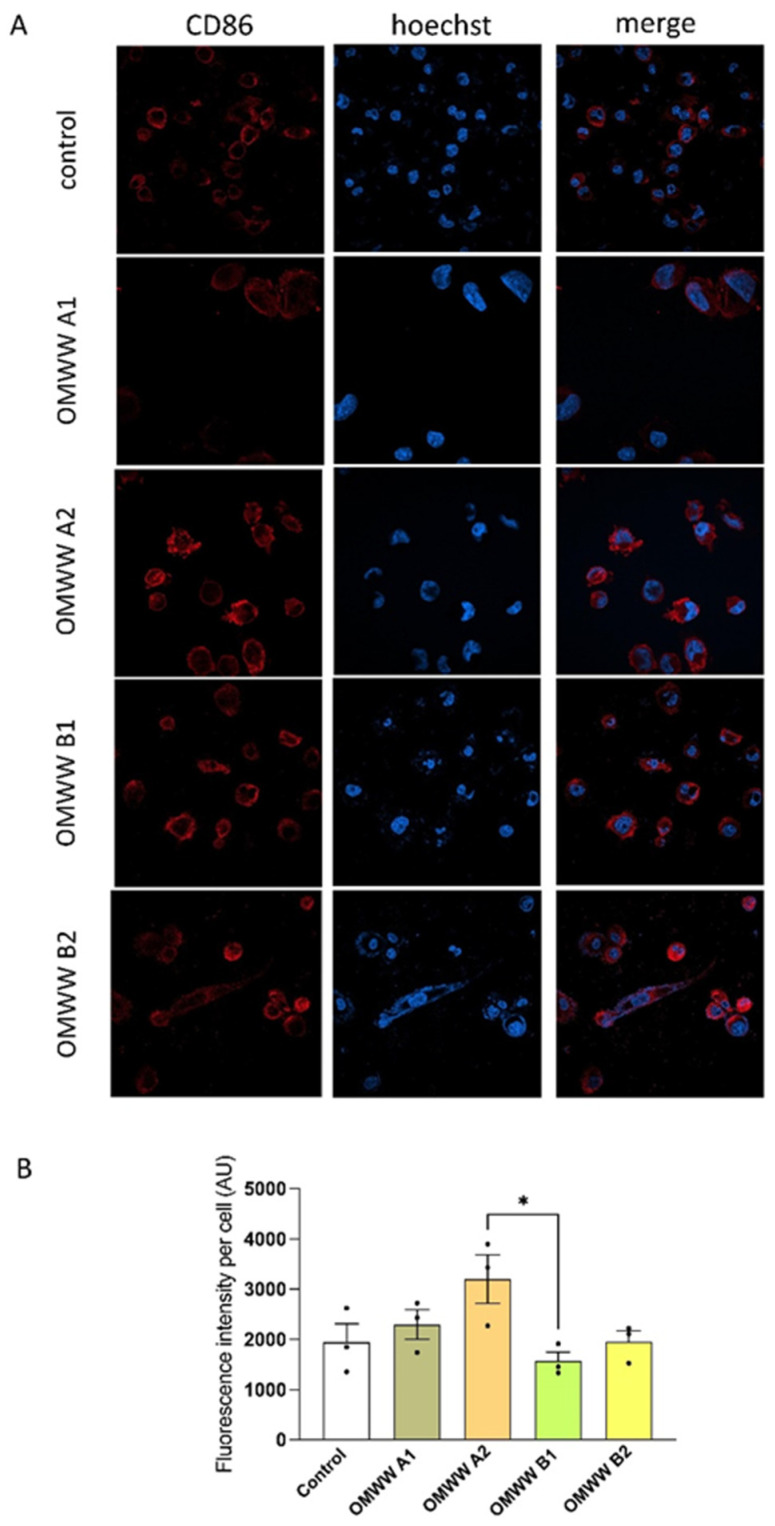
In vitro effect of OMWW exposure on THP-1-derived macrophage proinflammatory polarization, assessed as variation of CD86 protein expression by ICC. (**A**). Confocal images of THP-1-derived macrophages treated for 24 with OMWWs stained with Hoechst (blue, nuclei) and anti-CD86 (red). (**B**). Quantification of CD86 protein expression reported as AU of fluorescence intensity per cell. Data are reported as mean ± S.E.M. of three different experiments, each completed in triplicate. * *p* < 0.05.

**Figure 8:**
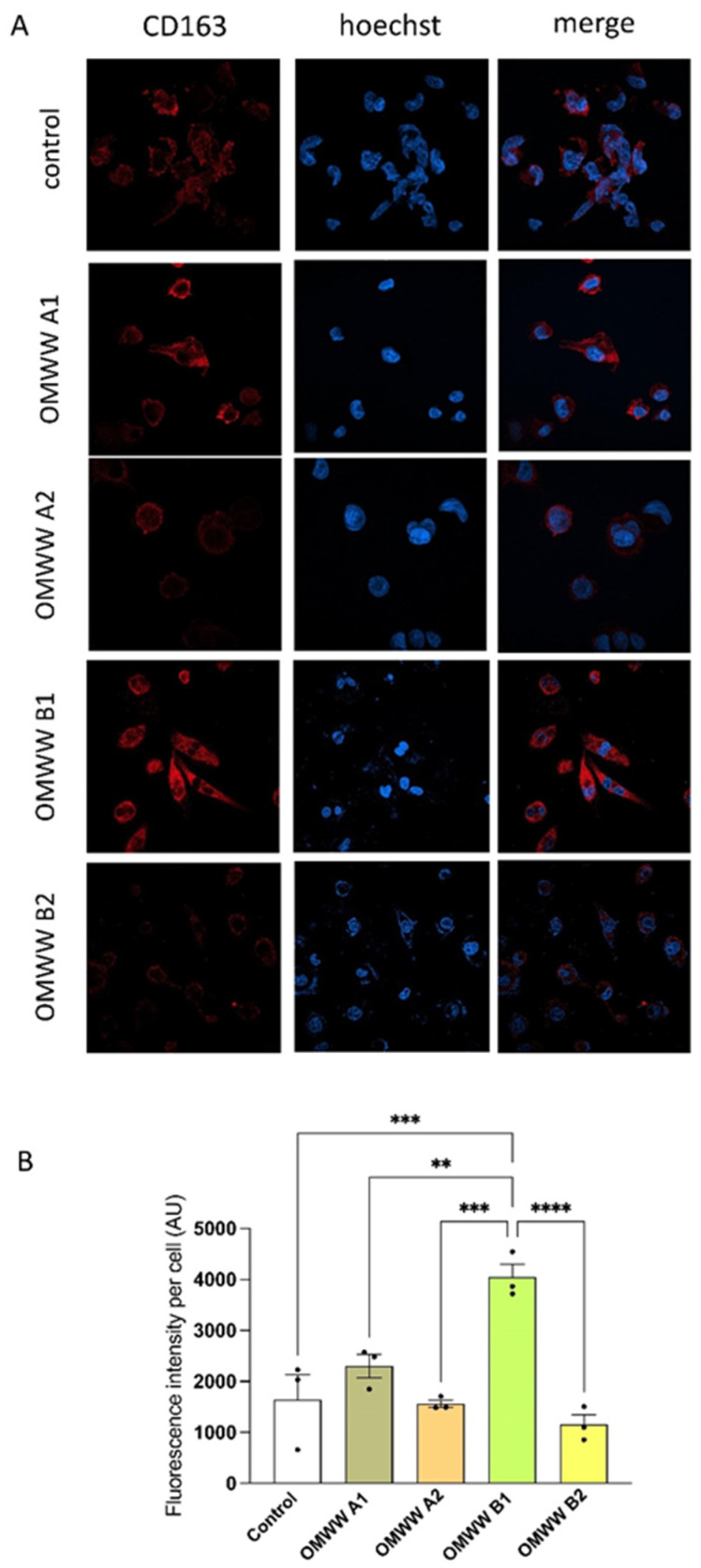
In vitro effect of OMWW exposure on THP-1-derived macrophage anti-inflammatory polarization, assessed as variation of CD163 protein expression by the ICC. (**A**). Confocal images of THP-1-derived macrophage treated for 24 with OMWWs stained with Hoechst (nuclei, blue) and anti-CD163 (red). (**B**). Quantification of CD163 protein expression was reported as AU of fluorescence intensity per cell. Data are reported as mean ± S.E.M. of three different experiments, each completed in triplicate. ** *p* < 0.01, *** *p* < 0.001, **** *p* < 0.001.

The anti-inflammatory effect of OMWWs was assessed on THP1-derived macrophages evaluating the protein expression of differentiation markers CD86, expressed in M1-like proinflammatory macrophages, and CD163, expressed in M2-like anti-inflammatory macrophages. As reported in Figure 7, OMWWs did not affect the expression of CD86, index of M1-like polarization, which in treated cells is comparable to the control medium-treated cells, indicating a lack of pro-inflammatory effect. At variance, OMWW B1 significantly increased the expression of CD163 with respect to untreated control cells (*p* < 0.001) and to all the other three OMWWs, promoting macrophage differentiation towards an anti-inflammatory M2-like phenotype (Figure 8). This effect was not observed with the other three OMWWs, probably due to the peculiar composition of OMWW B1, rich in oleacein. This hypothesis is supported by a variety of studies that demonstrated a positive effect of this specific polyphenol on both inflammation and oxidative stress [72,73]. These results suggest that OMWW B1 can exert an anti-inflammatory effect by acting on the macrophage phenotype.

## 4. Conclusions

The present work aimed at valorizing one of the principal EVOO production by-products, namely OMWW. Considering the high production of OMWWs nowadays (for 1 L of EVOO, 2.5 L of OMWWs are obtained), this work intended to evaluate the polyphenolic composition and the nutraceutical properties of different OMWW extracts. We investigated the variations in polyphenolic content and nutraceutical properties of OMWWs and related EVOOs derived from different cultivars and collected at diverse periods of the crushing season. The analytical characterization demonstrated a high polyphenolic content as well as antiradical activity for all the OMWW extracts when compared to related EVOO extracts, in particular, OMWW B1 showed the highest amount of polyphenols. In what concerns ROS, a remarkable efficacy to scavenge DPPH, ABTS, O_2_^−^, and HOCl^−^ was verified. A correlation of antiradical activity with polyphenolic content was found, demonstrating that OMWW B1 and B2 possess the strongest activity. Regarding cell viability, all samples did not present adverse effects on Caco-2 and HT29-MTX cells in concentrations below 10 μg/mL. Moreover, the intestinal permeation in an intestinal co-culture model composed of Caco-2 and HT29-MTX cell lines, a valuable model for investigating transport mechanisms across the intestinal epithelium, showed the permeation profile of representative OMWW polyphenols, with tyrosol achieving the best permeation rate (almost 60%). Furthermore, OMWW B1 demonstrated an anti-inflammatory involvement in THP macrophage monocyte cell lines. This peculiar effect of OMWW B1 on macrophages could be exploited for inflammatory disease of different aetiologies, affecting different organs, in which macrophage activation is involved. Therefore, the results of this study encourage further investigations to assess the reuse of early-stage OMWWs rich in polyphenols as a source of bioactive compounds with potential benefits in nutraceutical applications.

## Figures and Tables

**Table 1:** HPLC analysis of the main polyphenol (mg/kg of OMWW or EVOO) and total polyphenol content (TPC) obtained by Folin Ciocalteau (FC) method of OMWW and EVOO extracts. Values are expressed as mean ± standard deviation (n = 3).

Polyphenols mg/kg	OMWW- A1(CF)	EVOO- A1(CF)	OMWW- B1(CL)	EVOO- B1(CL)	OMWW- A2(CF)	EVOO- A2(CF)	OMWW- B2(CL)	EVOO- B2(CL)
	Early stage	Early stage	Early stage	Early stage	Later stage	Later stage	Later stage	Later stage
Hydroxytyrosol	92.4 ± 7.8 ^b^	0.99 ± 0.05 ^c^	172.7 ± 4.2 ^b^	1.38 ± 0.3 ^c^	190.7 ± 18 ^a^	4.2 ± 0.9 ^c^	339.0 ± 20.5 ^a^	1.5 ± 0.03 ^c^
Tyrosol	30.0 ± 1.8 ^e^	1.38 ± 0.2 ^c^	54.2 ± 1.0 ^c^	8.2 ± 0.01 ^c^	61.6 ±10 ^b^	3.2 ± 0.3 ^c^	89.6 ± 4.9 ^c^	10.6 ± 0.2 ^c^
Caffeic acid	16.2 ± 1.3 ^e,f^	nd	12.9 ± 0.8 ^d^	nd	59.4 ± 3.6 ^b^	nd	55.7 ± 9 ^d^	nd
Vanillic acid	4.1 ± 0.4 ^f^	nd	4.4 ± 0.02 ^d^	nd	5.8 ± 1.5 ^c^	nd	7.0 ± 1 ^e^	nd
Verbascoside	72.0 ± 6.9 ^c^	nd	190.1 ± 2.5 ^b^	nd	nd	nd	164.7 ± 27 ^b^	nd
Oleuropein	36.1 ± 3.4 ^e^	nd	15.1 ± 5.3 ^d^	nd	nd	nd	nd	nd
Pinoresinol	13.1 ± 3.7 ^f^	nd	36.6 ± 3.6 ^c,d^	nd	nd	nd	30.1 ± 3.2 ^d^	nd
Apigenin-7-glucoside	50.8 ± 1.5 ^d^	nd	96.8 ± 7.3 ^c^	nd	nd	nd	44.6 ± 0.2 ^d^	nd
Oleacein	153.8 ± 13 ^a^	49.3 ± 2.1 ^b^	672.6 ± 55.1 ^a^	157 ± 5.7 ^b^	nd	29.2 ± 2.1 ^b^	nd	180.7 ± 4.9 ^b^
Oleocanthal	nd	224.9 ± 14.7 ^a^	nd	369.4 ± 4.8 ^a^	nd	216 ± 11.7 ^a^	nd	379.6 ± 8 ^a^
Total phenols	455.5 ± 17.6	276.6 ± 14.8	1255.4 ± 56.17	536.0 ± 7.45	317.5 ± 20.9	252.6 ± 11.9	730.8 ± 35.6	572.4 ± 9.38
TPC FC	987.8 ± 29.1	307.6 ± 73.2	1529 ± 24.6	616.9 ± 3.00	457.8 ± 9.7	268 ± 30.8	1512.2 ± 23.6	609.1 ± 77

nd—not detected. Different letters (^a^, ^b^, ^c^, ^d^, ^e^, ^f^) in the same column indicate significant differences between mean values (*p* < 0.05).

**Table 2:** Antiradical scavenging capacity (IC_50_ mg/mL) of OMWW and EVOO phenolic extracts measured by DPPH and ABTS assays. Values are expressed as mean ± standard error (n = 3).

Samples	DPPH IC_50_ mg/mL	ABTS IC_50_ mg/mL
OMWW A1	0.792 ± 0.02 ^c^	0.325 ± 0.006 ^f^
OMWW A2	0.769 ± 0.05 ^c^	0.440 ± 0.02 ^e^
OMWW B1	0.165 ± 0.02 ^g^	0.216 ± 0.006 ^g^
OMWW B2	0.258 ± 0.007 ^f^	0.144 ± 0.01 ^h^
EVOO A1	1.16 ± 0.02 ^b^	1.83 ± 0.01 ^a^
EVOO A2	1.61± 0.02 ^a^	1.72 ± 0.05 ^b^
EVOO B1	0.47 ± 0.004 ^e^	0.73 ± 0.01 ^d^
EVOO B2	0.58 ± 0.009 ^d^	0.84 ± 0.02 ^c^
Trolox	0.007 ± 0.001 ^h^	0.003 ± 0.0004 ^i^

Different letters (^a^, ^b^, ^c^, ^d^, ^e^, ^f^, ^g^, ^h^, ^i^) in the same column indicate significant differences between mean values (*p* < 0.05).

**Table 3:** Superoxide anion radical (O_2_^−^) and hypochlorous acid (HOCl^−^) scavenging capacities of OMWW extracts. Values are expressed as mean ± standard deviation (n = 3).

Samples	ROS	
	O_2_^−^	HOCl^−^
	IC_50_ (μg/mL)	
OMWW A1	134.07 ± 17.44 ^a^	120.94 ± 9.99 ^a^
OMWW A2	48.76 ± 4.53 ^b,c^	113.88 ± 3.79 ^a^
OMWW B1	52.48 ± 1.58 ^b^	78.07 ± 3.04 ^a^
OMWW B2	40.07 ± 3.93 ^b,c^	96.34 ± 1.83 ^a^
Positive Controls		
Gallic acid	6.34 ± 0.21 ^d^	2.60 ± 0.05 ^b^
Catechin	18.01 ± 0.34 ^c,d^	0.20 ± 0.01 ^b^

Different letters (^a^, ^b^, ^c^, ^d^) in the same column indicate significant differences between mean values (*p* < 0.05).

**Table 4:** Mean permeation rates (%) at 240 min for all the detected polyphenols.

Analyte	Mean Permeation Rate (±SD) at 240 min (%)
Tyrosol	59 ± 29
Hydroxytyrosol	34 ± 5
Caffeic acid	26 ± 3
Vanillic acid	48 ± 2
Pinoresinol	44 ± 12

## Data Availability

The original contributions presented in the study are included in the article/supplementary material, further inquiries can be directed to the corresponding author.

## Supplementary Materials

The following supporting information can be downloaded at: https://www.mdpi.com/article/10.3390/foods13203312/s1, Figure S1: EVOO A1 Chromatograms; Figure S2: EVOO B1 Chromatograms; Figure S3: EVOO A2 Chromatograms; Figure S4: EVOO B2 Chromatograms; Figure S5: OMWW A1 Chromatograms; Figure S6: OMWW B1 Chromatograms; Figure S7: OMWW A2 Chromatograms; Figure S8: OMWW B2 Chromatograms; Table S1: Calculated concentrations of polyphenols in the basolateral and apical sides during the evaluation of compounds’ permeability across the barrier. Results are expressed as Mean ± SD (n = 3).

## References

[1] Garcia-Oliveira P., Jimenez-Lopez C., Lourenço-Lopes C., Chamorro F., Pereira A.G., Carrera-Casais A., Fraga-Corral M., Carpena M., Simal-Gandara J., Prieto M.A. (2021). Evolution of Flavors in Extra Virgin Olive Oil Shelf-Life. Antioxidants.

[2] Jimenez-Lopez C., Carpena M., Lourenço-Lopes C., Gallardo-Gomez M., Lorenzo J.M., Barba F.J., Prieto M.A., Simal-Gandara J. (2020). Bioactive Compounds and Quality of Extra Virgin Olive Oil. Foods.

[3] Nocella C., Cammisotto V., Fianchini L., D’Amico A., Novo M., Castellani V., Stefanini L., Violi F., Carnevale R. (2018). Extra Virgin Olive Oil and Cardiovascular Diseases: Benefits for Human Health. Endocr. Metab. Immune Disord. Drug Targets.

[4] Yubero-Serrano E.M., Lopez-Moreno J., Gomez-Delgado F., Lopez-Miranda J. (2019). Extra Virgin Olive Oil: More than a Healthy Fat. Eur. J. Clin. Nutr..

[5] Jiménez-Sánchez A., Martínez-Ortega A.J., Remón-Ruiz P.J., Piñar-Gutiérrez A., Pereira-Cunill J.L., García-Luna P.P. (2022). Therapeutic Properties and Use of Extra Virgin Olive Oil in Clinical Nutrition: A Narrative Review and Literature Update. Nutrients.

[6] Rana A., Samtiya M., Dhewa T., Mishra V., Aluko R.E. (2022). Health Benefits of Polyphenols: A Concise Review. J. Food Biochem..

[7] Virruso C., Accardi G., Colonna-Romano G., Candore G., Vasto S., Caruso C. (2014). Nutraceutical Properties of Extra-Virgin Olive Oil: A Natural Remedy for Age-Related Disease?. Rejuvenation Res..

[8] Barbalace M.C., Freschi M., Rinaldi I., Zallocco L., Malaguti M., Manera C., Ortore G., Zuccarini M., Ronci M., Cuffaro D. (2024). Unraveling the Protective Role of Oleocanthal and Its Oxidation Product, Oleocanthalic Acid, against Neuroinflammation. Antioxidants.

[9] Artajo L.-S., Romero M.-P., Suárez M., Motilva M.-J. (2007). Partition of Phenolic Compounds during the Virgin Olive Oil Industrial Extraction Process. Eur. Food Res. Technol..

[10] Borja R., Alba J., Banks C.J. (1997). Impact of the Main Phenolic Compounds of Olive Mill Wastewater (OMW) on the Kinetics of Acetoclastic Methanogenesis. Process. Biochem..

[11] Eroğlu E., Eroğlu İ., Gündüz U., Türker L., Yücel M. (2006). Biological Hydrogen Production from Olive Mill Wastewater with Two-Stage Processes. Int. J. Hydrogen Energy.

[12] Rinaldi M., Rana G., Introna M. (2003). Olive-Mill Wastewater Spreading in Southern Italy: Effects on a Durum Wheat Crop. Field Crops Res..

[13] De Marco E., Savarese M., Paduano A., Sacchi R. (2007). Characterization and Fractionation of Phenolic Compounds Extracted from Olive Oil Mill Wastewaters. Food Chem..

[14] Nakbi A., Dabbou S., Champion S., Fouchier F., Mehri S., Attia N., Leger C., Hammami M. (2011). Modulation of the Superoxide Anion Production and MMP-9 Expression in PMA Stimulated THP-1 Cells by Olive Oil Minor Components: Tyrosol and Hydroxytyrosol. Food Res. Int..

[15] Gabbia D., Carpi S., Sarcognato S., Zanotto I., Sayaf K., Colognesi M., Polini B., Digiacomo M., Macchia M., Nieri P. (2023). The Phenolic Compounds Tyrosol and Hydroxytyrosol Counteract Liver Fibrogenesis via the Transcriptional Modulation of NADPH Oxidases and Oxidative Stress-Related miRNAs. Biomed. Pharmacother..

[16] Elmaksoud H.A.A., Motawea M.H., Desoky A.A., Elharrif M.G., Ibrahimi A. (2021). Hydroxytyrosol Alleviate Intestinal Inflammation, Oxidative Stress and Apoptosis Resulted in Ulcerative Colitis. Biomed. Pharmacother..

[17] Franceschelli S., De Cecco F., Pesce M., Ripari P., Guagnano M.T., Nuevo A.B., Grilli A., Sancilio S., Speranza L. (2023). Hydroxytyrosol Reduces Foam Cell Formation and Endothelial Inflammation Regulating the PPARγ/LXRα/ABCA1 Pathway. Int. J. Mol. Sci..

[18] Fuccelli R., Fabiani R., Rosignoli P. (2018). Hydroxytyrosol Exerts Anti-Inflammatory and Anti-Oxidant Activities in a Mouse Model of Systemic Inflammation. Molecules.

[19] Zahi M.R., Zam W., El Hattab M. (2022). State of Knowledge on Chemical, Biological and Nutritional Properties of Olive Mill Wastewater. Food Chem..

[20] Huang Y., Guan Q., Zhang Z., Wang P., Li C. (2024). Oleacein: A Comprehensive Review of Its Extraction, Purification, Absorption, Metabolism, and Health Effects. Food Chem..

[21] Khalil A.A., Rahman M.M., Rauf A., Islam M.R., Manna S.J., Khan A.A., Ullah S., Akhtar M.N., Aljohani A.S.M., Abdulmonem W.A. (2024). Oleuropein: Chemistry, Extraction Techniques and Nutraceutical Perspectives-An Update. Crit. Rev. Food Sci. Nutr..

[22] Cuffaro D., Pinto D., Silva A.M., Bertolini A., Bertini S., Saba A., Macchia M., Rodrigues F., Digiacomo M. (2023). Insights into the Antioxidant/Antiradical Effects and In Vitro Intestinal Permeation of Oleocanthal and Its Metabolites Tyrosol and Oleocanthalic Acid. Molecules.

[23] Pereira C., Costa J., Sarmento B., Araújo F., Sarmento B. (2016). 3.3—Cell-Based in Vitro Models for Intestinal Permeability Studies. Concepts and Models for Drug Permeability Studies.

[24] Pinto D., Silva A.M., Dall’Acqua S., Sut S., Vallverdú-Queralt A., Delerue-Matos C., Rodrigues F. (2023). Simulated Gastrointestinal Digestion of Chestnut (*Castanea sativa* Mill.) Shell Extract Prepared by Subcritical Water Extraction: Bioaccessibility, Bioactivity, and Intestinal Permeability by In Vitro Assays. Antioxidants.

[25] Ferro M.D., Lopes E., Afonso M., Peixe A., Rodrigues F.M., Duarte M.F. (2020). Phenolic Profile Characterization of ‘Galega Vulgar’ and ‘Cobrançosa’ Portuguese Olive Cultivars along the Ripening Stages. Appl. Sci..

[26] Lozoya-Agullo I., Araújo F., González-Álvarez I., Merino-Sanjuán M., González-Álvarez M., Bermejo M., Sarmento B. (2017). Usefulness of Caco-2/HT29-MTX and Caco-2/HT29-MTX/Raji B Coculture Models To Predict Intestinal and Colonic Permeability Compared to Caco-2 Monoculture. Mol. Pharm..

[27] Palla M., Digiacomo M., Cristani C., Bertini S., Giovannetti M., Macchia M., Manera C., Agnolucci M. (2018). Composition of Health-Promoting Phenolic Compounds in Two Extra Virgin Olive Oils and Diversity of Associated Yeasts. J. Food Compos. Anal..

[28] Esposito Salsano J., Digiacomo M., Cuffaro D., Bertini S., Macchia M. (2022). Content Variations in Oleocanthalic Acid and Other Phenolic Compounds in Extra-Virgin Olive Oil during Storage. Foods.

[29] Cuffaro D., Bertolini A., Bertini S., Ricci C., Cascone M.G., Danti S., Saba A., Macchia M., Digiacomo M. (2023). Olive Mill Wastewater as Source of Polyphenols with Nutraceutical Properties. Nutrients.

[30] Alessandri S., Ieri F., Romani A. (2014). Minor Polar Compounds in Extra Virgin Olive Oil: Correlation between HPLC-DAD-MS and the Folin-Ciocalteu Spectrophotometric Method. J. Agric. Food Chem..

[31] Cuffaro D., Bertini S., Macchia M., Digiacomo M. (2023). Enhanced Nutraceutical Properties of Extra Virgin Olive Oil Extract by Olive Leaf Enrichment. Nutrients.

[32] Gomes A., Fernandes E., Silva A.M.S., Santos C.M.M., Pinto D.C.G.A., Cavaleiro J.A.S., Lima J.L.F.C. (2007). 2-Styrylchromones: Novel Strong Scavengers of Reactive Oxygen and Nitrogen Species. Bioorganic Med. Chem..

[33] Pinto D., Rodrigues F., Braga N., Santos J., Pimentel F.B., Palmeira-de-Oliveira A., Oliveira M.B.P.P. (2017). The Castanea Sativa Bur as a New Potential Ingredient for Nutraceutical and Cosmetic Outcomes: Preliminary Studies. Food Funct..

[34] González F., García-Martínez E., Del Mar Camacho M., Martínez-Navarrete N., Sarmento B., Fernandes I., Freitas V., Rodrigues F., Oliveira B. (2019). Insights into the Development of Grapefruit Nutraceutical Powder by Spray Drying: Physical Characterization, Chemical Composition and 3D Intestinal Permeability. J. Sci. Food Agric..

[35] Frión-Herrera Y., Gabbia D., Scaffidi M., Zagni L., Cuesta-Rubio O., De Martin S., Carrara M. (2020). Cuban Brown Propolis Interferes in the Crosstalk between Colorectal Cancer Cells and M2 Macrophages. Nutrients.

[36] Reboredo-Rodríguez P., Varela-López A., Forbes-Hernández T.Y., Gasparrini M., Afrin S., Cianciosi D., Zhang J., Manna P.P., Bompadre S., Quiles J.L. (2018). Phenolic Compounds Isolated from Olive Oil as Nutraceutical Tools for the Prevention and Management of Cancer and Cardiovascular Diseases. Int. J. Mol. Sci..

[37] Rigacci S., Stefani M. (2016). Nutraceutical Properties of Olive Oil Polyphenols. An Itinerary from Cultured Cells through Animal Models to Humans. Int. J. Mol. Sci..

[38] Shabir S., Ilyas N., Saeed M., Bibi F., Sayyed R.Z., Almalki W.H. (2023). Treatment Technologies for Olive Mill Wastewater with Impacts on Plants. Environ. Res..

[39] Criado-Navarro I., Ledesma-Escobar C.A., Parrado-Martínez M.J., Marchal-López R.M., Olmo-Peinado J.M., Espejo-Calvo J.A., Priego-Capote F. (2022). Monitoring the Partition of Bioactive Compounds in the Extraction of Extra Virgin Olive Oil. LWT.

[40] Mulinacci N., Romani A., Galardi C., Pinelli P., Giaccherini C., Vincieri F.F. (2001). Polyphenolic Content in Olive Oil Waste Waters and Related Olive Samples. J. Agric. Food Chem..

[41] Rodis P.S., Karathanos V.T., Mantzavinou A. (2002). Partitioning of Olive Oil Antioxidants between Oil and Water Phases. J. Agric. Food Chem..

[42] Yakhlef W., Arhab R., Romero C., Brenes M., de Castro A., Medina E. (2018). Phenolic Composition and Antimicrobial Activity of Algerian Olive Products and By-Products. LWT.

[43] El-Abbassi A., Kiai H., Hafidi A. (2012). Phenolic Profile and Antioxidant Activities of Olive Mill Wastewater. Food Chem..

[44] Jarboui R., Saber Azab M., Bilel H., Moustafa S.M.N. (2024). Antifungal Effect of Fresh and Stored Olive Mill Wastewater and Its Ethyl Acetate Extract against Plant Pathogenic Fungi. Plant Prot. Sci..

[45] Servili M., Baldioli M., Selvaggini R., Macchioni A., Montedoro G. (1999). Phenolic Compounds of Olive Fruit: One- and Two-Dimensional Nuclear Magnetic Resonance Characterization of Nüzhenide and Its Distribution in the Constitutive Parts of Fruit. J. Agric. Food Chem..

[46] Kiritsakis A.K., Kiritsakis K.A., Tsitsipas C.K. (2020). A Review of the Evolution in the Research of Antioxidants in Olives and Olive Oil during the Last Four Decades. J. Food Bioact..

[47] López-Huertas E., Lozano-Sánchez J., Segura-Carretero A. (2021). Olive Oil Varieties and Ripening Stages Containing the Antioxidants Hydroxytyrosol and Derivatives in Compliance with EFSA Health Claim. Food Chem..

[48] Carrara M., Kelly M.T., Munier S., Paradis C., Belmiloudi S., Margout-Jantac D. (2024). Validation of Simple UPLC-MS-UV and HPLC-Fluorescence Methods for the Determination of Oleacein in Olive Mill Wastewater. Application in the Analysis of Oleacein in French Cultivars. ACS Food Sci. Technol..

[49] Silvan J.M., Pinto-Bustillos M.A., Vásquez-Ponce P., Prodanov M., Martinez-Rodriguez A.J. (2019). Olive Mill Wastewater as a Potential Source of Antibacterial and Anti-Inflammatory Compounds against the Food-Borne Pathogen *Campylobacter*. Innov. Food Sci. Emerg. Technol..

[50] Charoenprasert S., Mitchell A. (2012). Factors Influencing Phenolic Compounds in Table Olives (*Olea europaea*). J. Agric. Food Chem..

[51] Sivakumar G., Briccoli Bati C., Uccella N. (2005). HPLC-MS Screening of the Antioxidant Profile of Italian Olive Cultivars. Chem. Nat. Compd..

[52] Ryan D., Robards K., Lavee S. (1999). Changes in Phenolic Content of Olive during Maturation. Int. J. Food Sci. Technol..

[53] Cecchi L., Migliorini M., Cherubini C., Giusti M., Zanoni B., Innocenti M., Mulinacci N. (2013). Phenolic Profiles, Oil Amount and Sugar Content during Olive Ripening of Three Typical Tuscan Cultivars to Detect the Best Harvesting Time for Oil Production. Food Res. Int..

[54] Dali I., Abdelwahab A.T., Aydi A., Fares N., Eladeb A., Hamzaoui M., Abderrabba M., Abdelfattah M.A., Guetat A. (2023). Valorization of Lyophilized Olive Mill Wastewater: Chemical and Biochemical Approaches. Sustainability.

[55] Pandey K.B., Rizvi S.I. (2009). Plant Polyphenols as Dietary Antioxidants in Human Health and Disease. Oxid. Med. Cell. Longev..

[56] Gülçin I. (2006). Antioxidant Activity of Caffeic Acid (3,4-Dihydroxycinnamic Acid). Toxicology.

[57] Zhang B., Pan C., Feng C., Yan C., Yu Y., Chen Z., Guo C., Wang X. (2022). Role of Mitochondrial Reactive Oxygen Species in Homeostasis Regulation. Redox Rep..

[58] Di Meo S., Reed T.T., Venditti P., Victor V.M. (2016). Role of ROS and RNS Sources in Physiological and Pathological Conditions. Oxid. Med. Cell. Longev..

[59] Cardinali A., Pati S., Minervini F., D’Antuono I., Linsalata V., Lattanzio V. (2012). Verbascoside, Isoverbascoside, and Their Derivatives Recovered from Olive Mill Wastewater as Possible Food Antioxidants. J. Agric. Food Chem..

[60] Galano A., Alvarez-Idaboy J.R., Francisco-Márquez M., Medina M.E. (2012). A Quantum Chemical Study on the Free Radical Scavenging Activity of Tyrosol and Hydroxytyrosol. Theor. Chem. Acc..

[61] Czerwińska M., Kiss A.K., Naruszewicz M. (2012). A Comparison of Antioxidant Activities of Oleuropein and Its Dialdehydic Derivative from Olive Oil, Oleacein. Food Chem..

[62] Posadino A.M., Cossu A., Giordo R., Piscopo A., Abdel-Rahman W.M., Piga A., Pintus G. (2021). Antioxidant Properties of Olive Mill Wastewater Polyphenolic Extracts on Human Endothelial and Vascular Smooth Muscle Cells. Foods.

[63] Quirós-Sauceda A.E., Palafox-Carlos H., Sáyago-Ayerdi S.G., Ayala-Zavala J.F., Bello-Perez L.A., Alvarez-Parrilla E., de la Rosa L.A., González-Córdova A.F., González-Aguilar G.A. (2014). Dietary Fiber and Phenolic Compounds as Functional Ingredients: Interaction and Possible Effect after Ingestion. Food Funct..

[64] Jiménez-Moreno N., Cimminelli M.J., Volpe F., Ansó R., Esparza I., Mármol I., Rodríguez-Yoldi M.J., Ancín-Azpilicueta C. (2019). Phenolic Composition of Artichoke Waste and Its Antioxidant Capacity on Differentiated Caco-2 Cells. Nutrients.

[65] Wu T., Lv H., Wang F., Wang Y. (2016). Characterization of Polyphenols from Lycium Ruthenicum Fruit by UPLC-Q-TOF/MS(E) and Their Antioxidant Activity in Caco-2 Cells. J. Agric. Food Chem..

[66] Silva A.M., Pinto D., Moreira M.M., Costa P.C., Delerue-Matos C., Rodrigues F. (2022). Valorization of Kiwiberry Leaves Recovered by Ultrasound-Assisted Extraction for Skin Application: A Response Surface Methodology Approach. Antioxidants.

[67] López-Yerena A., Pérez M., Vallverdú-Queralt A., Miliarakis E., Lamuela-Raventós R.M., Escribano-Ferrer E. (2021). Oleacein Intestinal Permeation and Metabolism in Rats Using an In Situ Perfusion Technique. Pharmaceutics.

[68] Vitali Čepo D., Radić K., Turčić P., Anić D., Komar B., Šalov M. (2020). Food (Matrix) Effects on Bioaccessibility and Intestinal Permeability of Major Olive Antioxidants. Foods.

[69] Nieto J.A., Fernández-Jalao I., Siles-Sánchez M.D.L.N., Santoyo S., Jaime L. (2023). Implication of the Polymeric Phenolic Fraction and Matrix Effect on the Antioxidant Activity, Bioaccessibility, and Bioavailability of Grape Stem Extracts. Molecules.

[70] Zhang Y., Zou J., Chen R. (2022). An M0 Macrophage-Related Prognostic Model for Hepatocellular Carcinoma. BMC Cancer.

[71] Locati M., Curtale G., Mantovani A. (2020). Diversity, Mechanisms, and Significance of Macrophage Plasticity. Annu. Rev. Pathol..

[72] Filipek A., Mikołajczyk T.P., Guzik T.J., Naruszewicz M. (2020). Oleacein and Foam Cell Formation in Human Monocyte-Derived Macrophages: A Potential Strategy Against Early and Advanced Atherosclerotic Lesions. Pharmaceuticals.

[73] Filipek A., Czerwińska M.E., Kiss A.K., Wrzosek M., Naruszewicz M. (2015). Oleacein Enhances Anti-Inflammatory Activity of Human Macrophages by Increasing CD163 Receptor Expression. Phytomedicine.

