# Supplementary material for: Comparative Analysis on Polyphenolic Composition of Different Olive Mill Wastewater and Related Extra Virgin Olive Oil Extracts and Evaluation of Nutraceutical Properties by Cell-Based Studies
Source: Foods. 2024 Oct 18;13(20):3312. doi: 10.3390/foods13203312 (PMC11507932; doi:10.3390/foods13203312)
Supplement: Supplementary file 1 [file foods-13-03312-s001.zip › foods-3252548-supplementary.pdf]

Comparative Analysis on polyphenolic composition of different Olive Mill Wastewater and Related Extra Virgin Olive Oil Extracts and evaluation of nutraceutical properties by cell-based studies

Doretta Cuffaro [a,b], Andrea Bertolini [c], Ana Margarida Silva [d], Francisca Rodrigues [d], Daniela Gabbia [e], Sara De Martin [e], Alessandro Saba [b,c,f], Simone Bertini [a], Maria Digiacomo [a,b,f*] and Marco Macchia [a,b]

[a] Department of Pharmacy, University of Pisa, Via Bonanno 6, 56126 Pisa, Italy; doretta.cuffaro@unipi.it (D.C.); simone.bertini@unipi.it (S.B.); marco.macchia@unipi.it (M.M.)
[b] Interdepartmental Research Center "Nutraceuticals and Food for Health", University of Pisa, 56100 Pisa, Italy
[c] Department of Surgical, Medical and Molecular Pathology and Critical Care Area, University of Pisa, 56126 Pisa, Italy; a.bertolini@med.unipi.it (A.B.); alessandro.saba@unipi.it (A.S.)
[d] REQUIMTE/LAQV, ISEP, Polytechnic Institute of Porto, Rua Dr. António Bernardino de Almeida, 4249-015 Porto, Portugal; ana.silva@graq.isep.ipp.pt (A.M.S.); francisca.rodrigues@graq.isep.ipp.pt (F.R.)
[e] Department of Pharmaceutical and Pharmacological Sciences, University of Padova, 351131 Padova, Italy; daniela.gabbia@unipd.it (D.G.); sara.demartin@unipd.it (S.D.)
[f] Center for Instrument Sharing of the University of Pisa (CISUP), 56126 Pisa, Italy
* Correspondence: maria.digiacomo@unipi.it; Tel.: +39-050-2219594

Figure S1-S8 HPLC DAD representative chromatograms of EVOO and OMWW samples (IS: Internal Standard, 1= hydroxytyrosol, 2= tyrosol, 3= caffeic acid, 4=vanillic acid, 5= verbascoside, 6=oleuropein, 7=pinoresinol, 8=apigenin-7-glucoside, 9=oleacein, 10=oleocanthal.

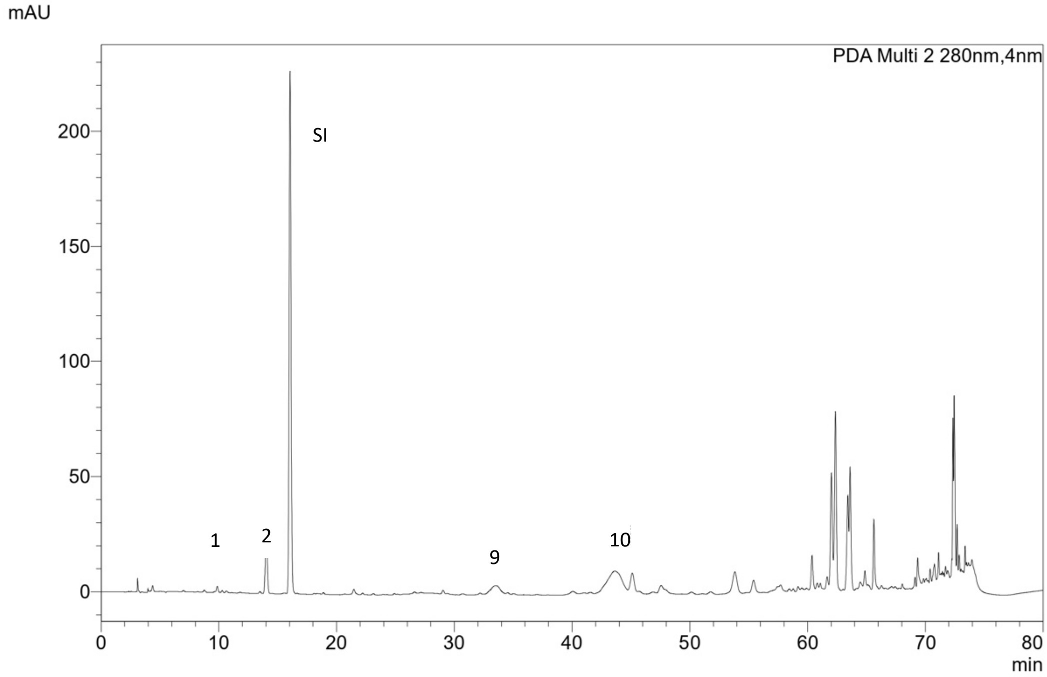

Figure S1. EVOO A1 Chromatograms.

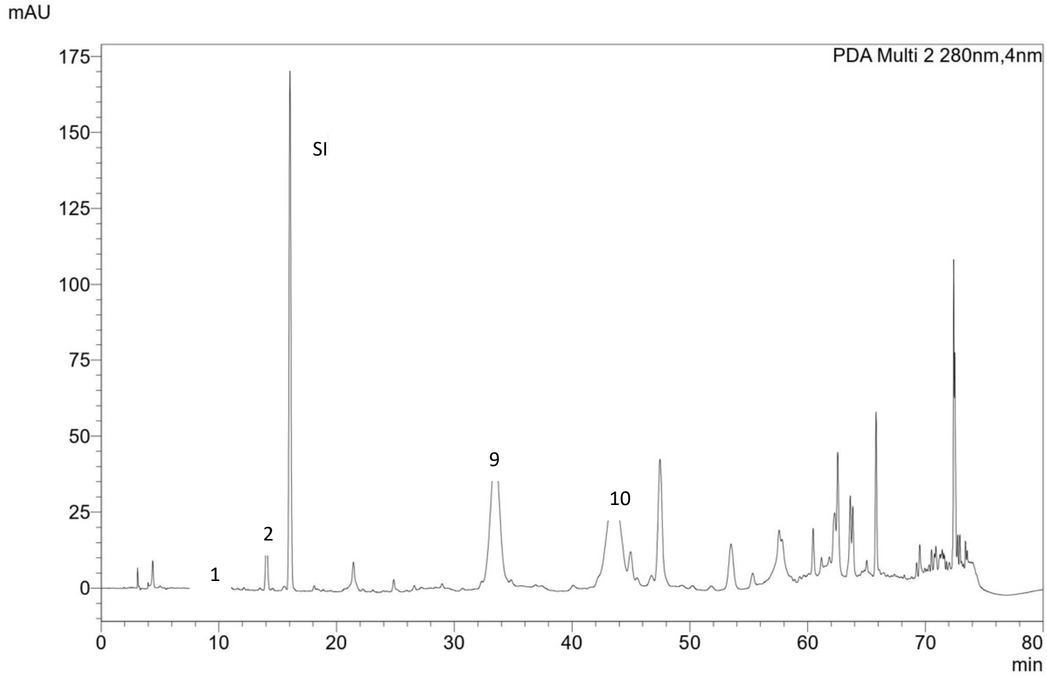

Figure S2. EVOO B1 Chromatograms

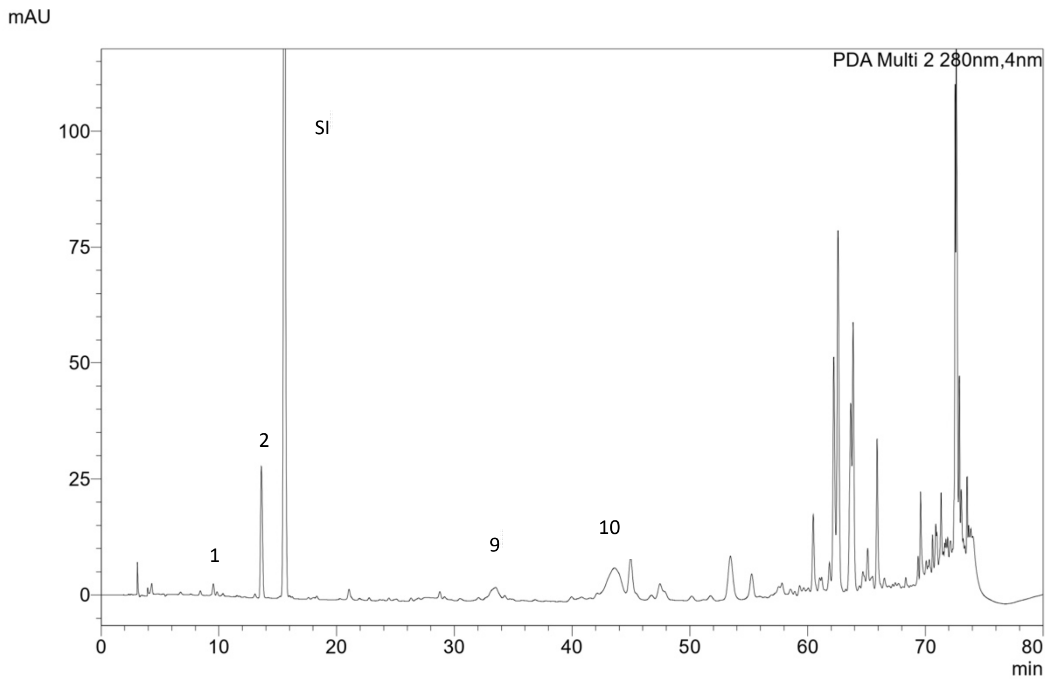

Figure S3. EVOO A2 Chromatograms

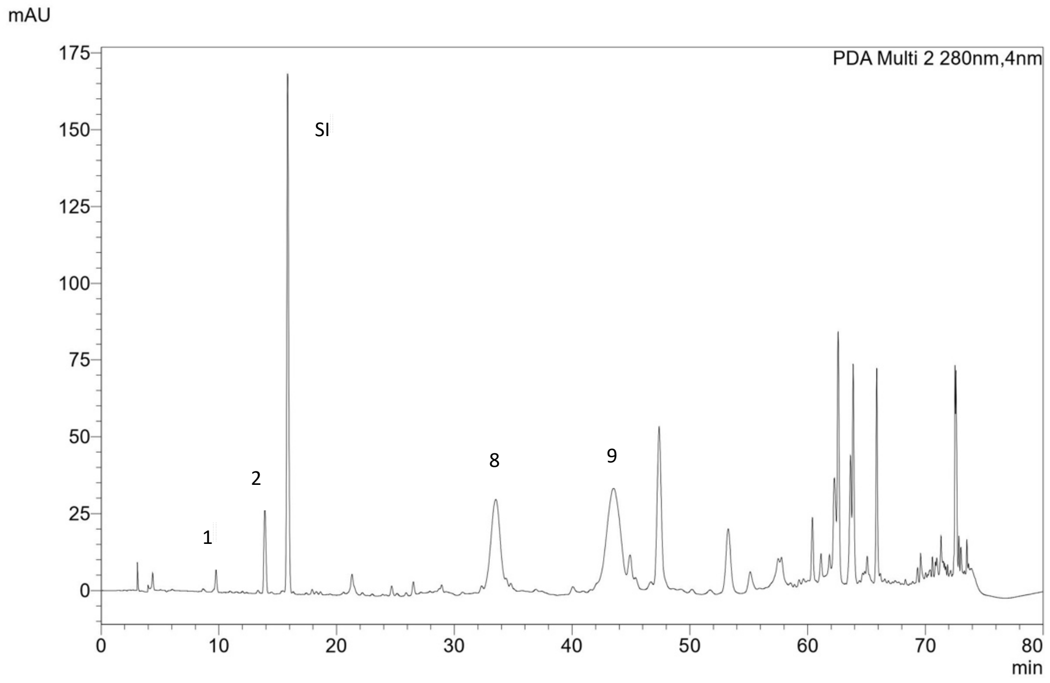

Figure S4. EVOO B2 Chromatograms

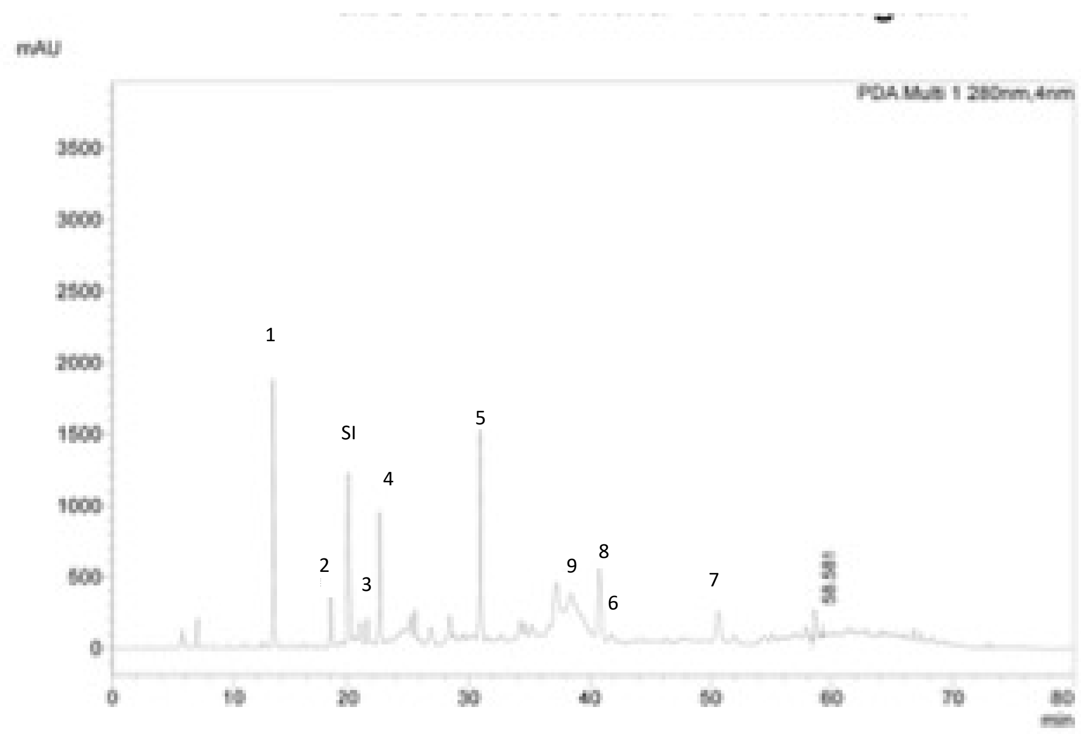

Figure S5. OMWW A1 Chromatograms

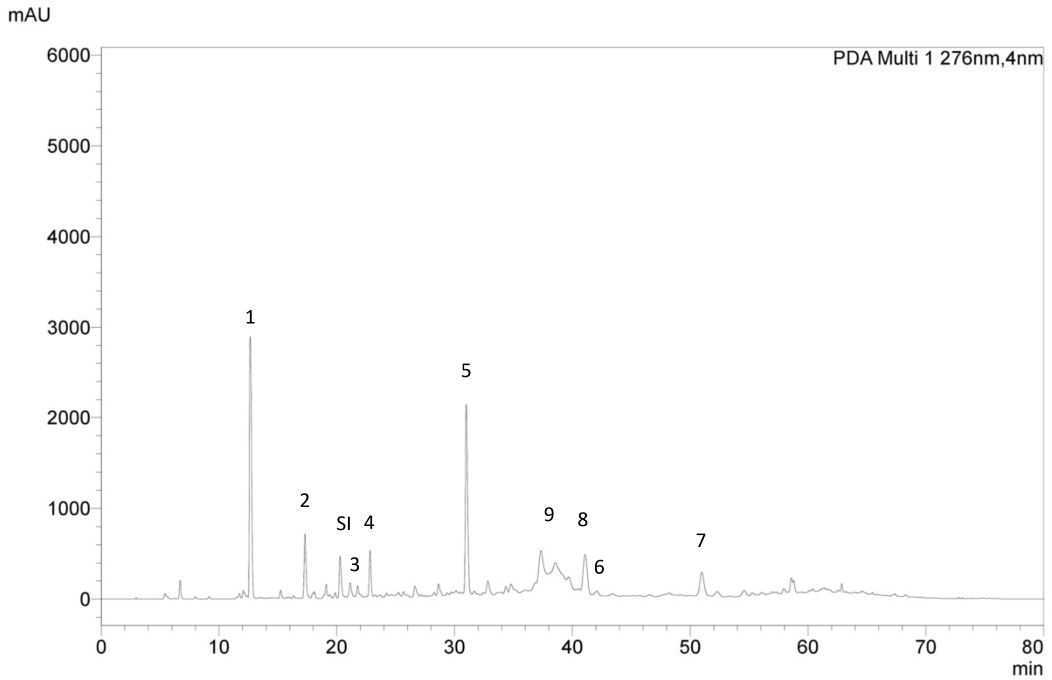

Figure S6. OMWW B1 Chromatograms

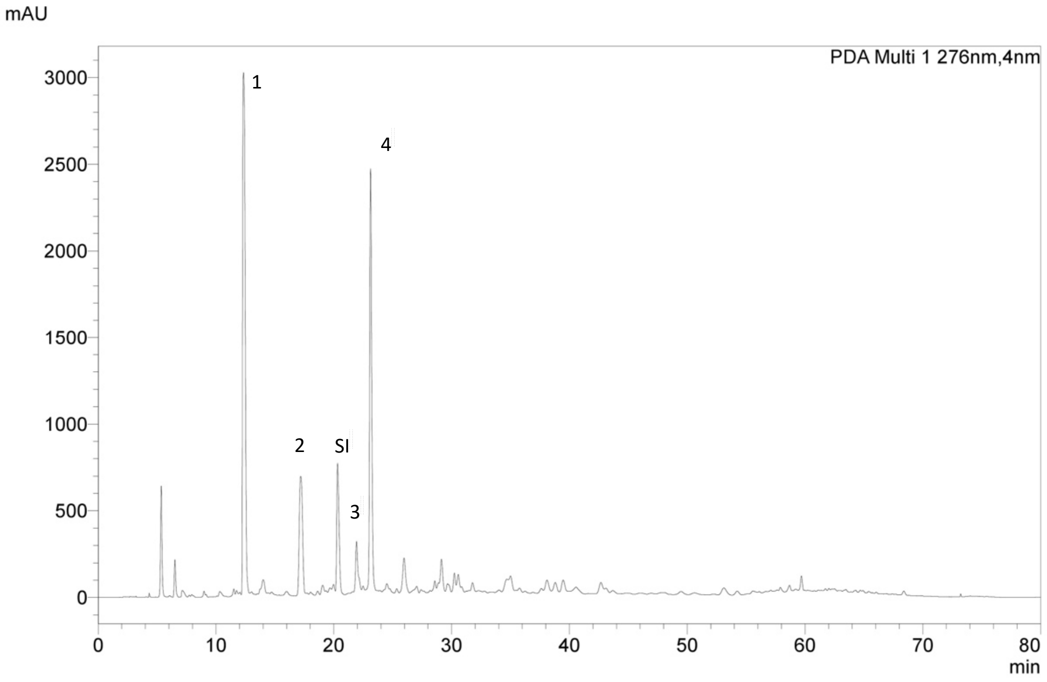

Figure S7. OMWW A2 Chromatograms

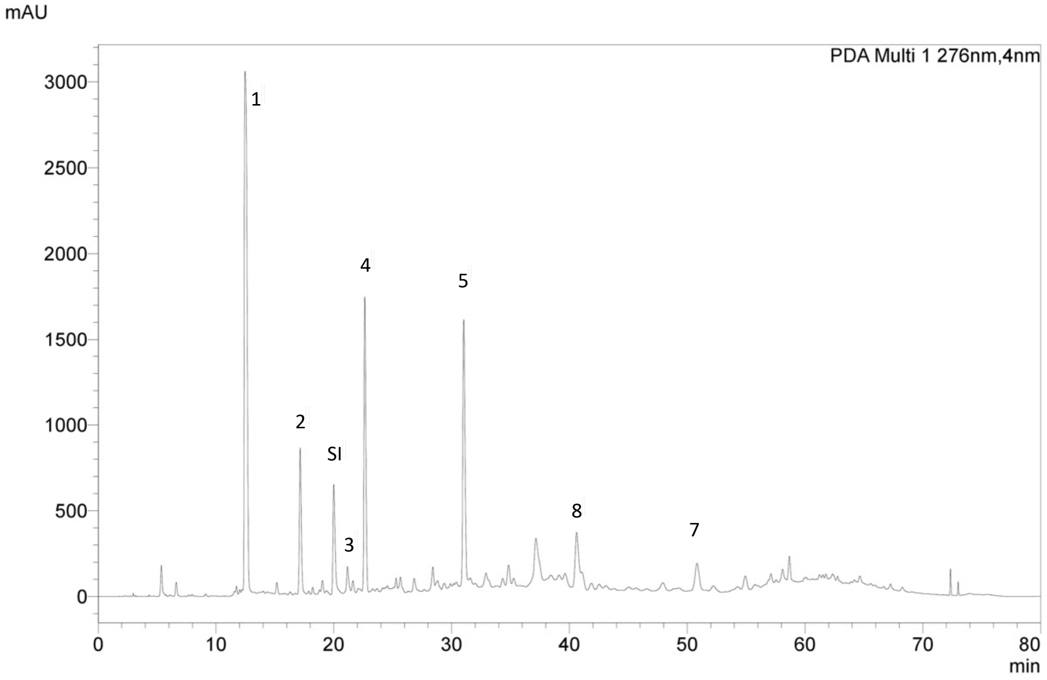

Figure S8. OMWW B2 Chromatograms

Table S1. Calculated concentrations of polyphenols in the basolateral and apical sides during the evaluation of compounds' permeability across the barrier. Results are expressed as Mean ± SD (n =3).

Time (min)	Tyrosol (ng/mL)	Hydroxytyrosol (ng/mL)	Caffeic Acid (ng/mL)	Vanillic Acid (ng/mL)	Pinoresinol (ng/mL)
**30**	155,50 ± 6,36	7,42 ± 5,35	0,31 ± 0,10	35,20 ± 1,41	3,83 ± 0,06
**60**	224,50 ± 21,92	9,12 ± 5,78	0,40 ± 0,25	45,50 ± 3,82	4,74 ± 0,21
**120**	259,50 ± 47,38	15,75 ± 5,02	1,29 ± 0,31	62,10 ± 2,55	6,33 ± 0,18
**180**	256,50 ± 13,44	19,40 ± 2,55	2,00 ± 0,01	72,70 ± 0,85	6,44 ± 2,76
**240**	314,00 ± 33,94	15,20 ± 1,98	1,89 ± 0,42	82,30 ± 4,81	8,57 ± 0,06
**Apical**	587,50 ± 234,05	45,30 ± 0,85	7,31 ± 0,74	170 ± 15,56	20,00 ± 5,37
